# Efficient Machine Learning Prediction of Solvent‐Dependent NMR Chemical Shifts in Zinc Complexes

**DOI:** 10.1002/jcc.70368

**Published:** 2026-04-12

**Authors:** Jyothika R. Pillay, Michael Ringleb, Alexander Croy, Stefan Zechel, Ulrich S. Schubert, Stefanie Gräfe

**Affiliations:** ^1^ Institute of Physical Chemistry (IPC) Friedrich Schiller University Jena Jena Germany; ^2^ Institute for Organic and Macromolecular Chemistry (IOMC) Friedrich Schiller University Jena Jena Germany; ^3^ Jena Center for Soft Matter (JCSM) Friedrich Schiller University Jena Jena Germany; ^4^ Helmholtz Institute for Polymers in Energy Applications (HIPOLE Jena) Jena Germany; ^5^ Helmholtz‐Zentrum Berlin Für Materialien und Energie (HZB) Berlin Germany; ^6^ Center for Energy and Environmental Chemistry Jena (CEEC Jena) Friedrich Schiller University Jena Jena Germany; ^7^ Fraunhofer Institute for Applied Optics and Precision Engineering (IOF) Jena Germany

**Keywords:** machine learning, NMR spectroscopy, SOAP descriptors, solvent effects, zinc complexes

## Abstract

Accurate prediction of NMR chemical shifts in transition metal complexes remains challenging due to the wide range of coordination environments and complex electronic structures of these systems. In this work, we present a machine learning approach (ML) for rapid and accurate prediction of 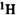 NMR shifts in zinc complexes across multiple solvent environments. We systematically selected a diverse set of zinc complexes from the transition metal quantum mechanics (tmQM) database using K‐means clustering on SOAP descriptors, and performed DFT NMR calculations across five solvents to generate training data. We combine smooth overlap of atomic positions (SOAP) descriptors with tree‐based ensemble methods to predict proton chemical shifts. Among several ML algorithms evaluated, LightGBM achieved the best performance on held‐out test complexes (MAE = 0.016 ppm, RMSE = 0.028 ppm, $R^{2}$ = 0.99), demonstrating excellent generalization to unseen molecular structures. External validation against experimental NMR data across multiple solvents revealed strong predictive performance ($R^{2}$ = 0.90, MAE = 0.56 ppm), with exceptional accuracy in methanol ($R^{2}$ = 0.96) and acetonitrile ($R^{2}$ = 0.91). Notably, the model demonstrated robust transferability to acetonitrile despite this solvent not being included in the training set. This approach provides a computationally efficient alternative to expensive quantum chemical calculations for predicting 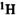 NMR shifts in transition metal complexes, offering prediction times that are orders of magnitude faster while maintaining accuracy comparable to DFT methods, potentially accelerating the characterization and design of organometallic compounds.

## Graphical abstract

Machine learning approach (ML) for rapid and accurate prediction of 1H‐NMR shifts in zinc complexes validated against experimental NMR data across multiple solvent environments.

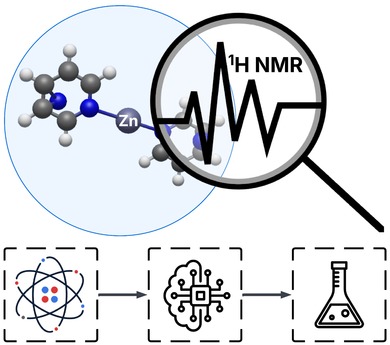

## 1 Introduction

Nuclear magnetic resonance (NMR) spectroscopy has emerged as an indispensable analytical tool for structural elucidation, dynamics, and reactivity in both organic and inorganic chemistry [1, 2]. For transition metal complexes, NMR spectroscopy provides crucial insights into the metal‐ligand interactions, coordination environments, and electronic structures [3, 4]. Despite its widespread application, the interpretation of NMR spectra for transition metal complexes remains challenging due to complex coupling patterns, quadrupolar effects, and paramagnetic contributions [5, 6, 7]. This challenge is further complicated by solvent effects, which can significantly influence the observed chemical shifts through dipolar interactions, hydrogen bonding, and changes in the molecular geometry [8, 9]. Additionally, the solvent can also counteract the complexation, as polar solvents like water can potentially act as better ligands compared to very weak complexing agents, leading to competitive binding and altered chemical environments [10, 11]. Therefore, the influence of solvation on NMR chemical shifts is particularly important for accurate interpretation of NMR data and for designing complexes with specific properties [12].

In recent decades, density functional theory (DFT) has proven to be effective in calculating NMR chemical shifts and magnetic shielding tensors in coordination chemistry [13, 14]. In particular, the gauge‐including atomic orbital (GIAO) method has emerged as a reliable approach for calculating these NMR properties in a variety of chemical environments [15]. Furthermore, the development of specialized functionals, relativistic corrections, and improved treatment of electron correlation has significantly enhanced the accuracy of DFT for NMR parameters across a wide range of chemical systems [16, 17, 18]. To account for solvent effects, implicit solvent models like the conductor‐like screening model (COSMO) [19] offer an efficient method for capturing solute–solvent interactions. Despite these advances, the high computational cost of advanced quantum chemical calculations limits the efficient exploration of the vast transition‐metal chemical space [20]. Furthermore, the selection of appropriate functionals, basis sets, and relativistic treatments requires significant expertise and often involves system‐specific optimization, with transition metals being particularly challenging, limiting the practical application of these methods for high‐throughput studies of solvent effects on NMR properties [21, 22, 23, 24, 25].

To address these computational challenges, machine learning (ML) approaches offer an attractive alternative by providing accurate predictions at a fraction of the computational cost of first‐principles methods [26, 27]. Recent advances in ML have shown promising results for predicting NMR chemical shifts of organic molecules, nucleic acids and proteins [28, 29, 30, 31, 32, 33, 34, 35, 36, 37]. For solid‐state systems, Paruzzo et al. developed ShiftML as a kernel‐based ML framework that successfully predicts ^1^H and ^13^C chemical shifts with DFT accuracy but at a fraction of the computational cost [38]. In their recent work, Büning and Grimme introduced a Δ‐ML approach that combined relatively inexpensive DFT calculations with ML corrections [39]. This hybrid method achieved CCSD(T)‐quality NMR chemical shifts, reducing mean absolute errors from 0.31 ppm with DFT to just 0.13 ppm for 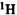 NMR shifts across a diverse test set. Ondar et al. successfully combined semiempirical modeling with machine learning to predict 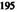
Pt‐NMR chemical shifts in organometallic complexes [40]. The emergence of artificial intelligence methods has significantly advanced the field of NMR chemical shift prediction, enabling more accurate and efficient modeling of complex molecular systems. Specifically, modern deep learning architectures—such as convolutional neural networks (CNNs), message passing neural networks (MPNNs), graph neural networks (GNNs), and transfer learning techniques—have demonstrated superior accuracy and computational efficiency compared to standard DFT approaches for NMR shift prediction [41, 42, 43, 44, 45, 46, 47, 48]. Despite these advances, predicting accurate NMR chemical shifts for transition metal complexes remains challenging due to insufficient training data and incomplete modeling of metal‐ligand interactions, highlighting an important area for potential advancement of ML approaches [49].

While large datasets are often associated with successful ML applications, generating extensive training data using DFT remains computationally demanding, particularly when implicit solvation models are included [50, 51]. For transition‐metal complexes, a single NMR calculation employing appropriate exchange‐correlation functionals and basis sets can require substantial computational resources, rendering exhaustive calculations across thousands of structures prohibitively expensive [14, 52]. As a result, strategies that rely on carefully selected training sets that balance chemical diversity with computational feasibility, are essential for developing reliable ML models for transition metal NMR prediction [49, 53]. Systematic clustering‐based selection methods using molecular descriptors offer an approach to identify representative structures that span the chemical space of interest. This approach has previously been shown to effectively select representative chemical structures from large chemical databases [54]. Such methods enable the development of accurate ML models with computationally feasible training set sizes.

Among transition metals, neutral zinc complexes offer significant advantages for NMR shift prediction. First, they exhibit a more consistent spectroscopic behavior compared to charged species due to the absence of counter‐ion effects [25, 55]. The ${\text{d}}^{10}$ electronic configuration of zinc(II) in these neutral complexes provides a diamagnetic environment that minimizes paramagnetic contributions to the NMR shielding tensors, allowing for more straightforward interpretation of solvent‐dependent spectral changes [56, 57, 58]. Additionally, zinc complexes exhibit variable coordination numbers and geometries depending on the nature of ligands and solvent environment, making them excellent probes for studying structure‐property relationships [59]. Furthermore, their widespread relevance in catalysis, biological systems, and materials science makes them valuable targets for computational modeling [60, 61, 62, 63, 64].

In this work, we address the challenge of predicting 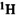 NMR shifts in zinc(II) complexes across various solvent environments using a computationally efficient ML framework. By employing a systematic clustering‐based selection strategy on a large database of zinc complexes, we identify a representative subset that captures the structural diversity while maintaining computational feasibility for DFT calculations. Our results demonstrate that tree‐based ensemble methods, particularly LightGBM, achieve exceptional prediction accuracy, with a test MAE of 0.016 ppm on unseen complexes. The trained models are further validated against experimental NMR data across multiple solvent environments, confirming their reliability and transferability. This work establishes a computationally efficient framework for high‐throughput NMR shift prediction in transition metal complexes, demonstrating that strategic data selection combined with ML methods enables accurate prediction of spectroscopic properties for coordination compounds at a fraction of the computational cost of quantum chemical calculations.

## 2 Methods

**FIGURE 1:**
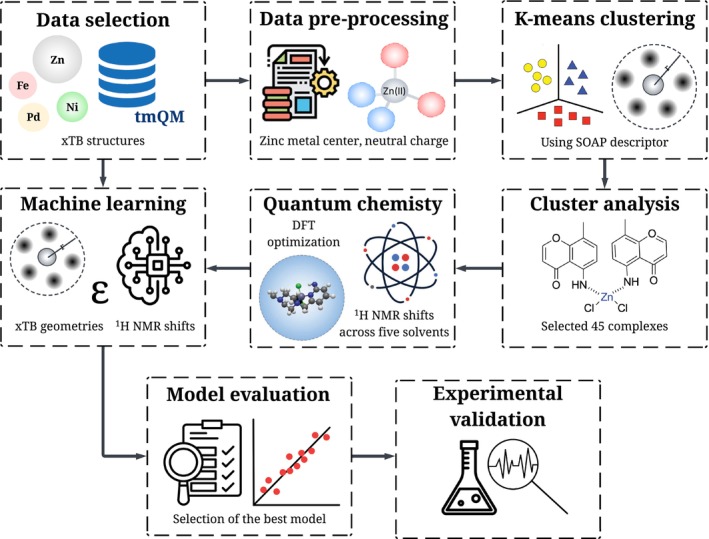
Computational workflow for machine learning‐based prediction of 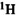 NMR chemical shifts in zinc complexes. The workflow begins with dataset selection from the tmQM database, yielding 5331 neutral zinc complexes, followed by SOAP descriptor‐based clustering to select 45 representative complexes. DFT calculations generate NMR shifts across five solvent environments, which are used to train and evaluate multiple ML algorithms. The trained model is validated against experimental data from 30 additional complexes.

To implement our study of solvent effects on 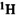 NMR shifts, we established a computational protocol with five steps: (1) Dataset curation from the transition metal quantum mechanics (tmQM) dataset [65], (2) clustering‐based selection of representative structures using smooth overlap of atomic positions (SOAP) [66] descriptor, (3) DFT‐based NMR calculations in five solvent environments, (4) comparative evaluation of machine learning algorithms for the prediction of 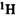 NMR shifts, and (5) validation against experimental 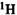 NMR shifts to confirm predictive accuracy (Figure 1).

### 2.1 Data Selection and Pre‐Processing

We selected structures from the tmQM dataset for our computational study. All structures in the tmQM dataset are already optimized at the GFN2‐xTB level [67]. The dataset was filtered to include only neutral zinc complexes, resulting in a subset of 5331 complexes. This selection criterion was applied to minimize computational artifacts that arise from modeling charged species in different solvent environments [68].

### 2.2 Clustering and Representative Sampling

To design a computationally efficient yet structurally representative subset of zinc complexes, K‐means clustering [69] was applied to the 5331 neutral complexes. The atomic structure of the complexes was represented using the smooth overlap of atomic positions (SOAP) descriptor [66], calculated with the DScribe package [70]. The SOAP descriptor provides a comprehensive molecular representation of the local atomic environments through the expansion of atomic densities in terms of radial basis functions and spherical harmonics [66]. For each atom i in a molecule, the SOAP descriptor is computed by:

(1) $$p_{i}^{Z_{1},Z_{2}}(n,l,m,m^{\prime })=\underset{\alpha ,\beta }{\sum }c_{\alpha }^{Z_{1}}c_{\beta }^{Z_{2}}\int drg_{n}(r)Y_{lm}(\hat{r})g_{n^{\prime }}(r)Y_{lm^{\prime }}(\hat{r}),$$

where $g_{n}(r)$ represents the radial basis functions, $Y_{lm}(\hat{r})$ are the spherical harmonics, and $c_{\alpha }^{Z}$ are the expansion coefficients for atoms of type Z.

To visualize the distribution of complexes in chemical space, a subsequent dimensionality reduction using t‐distributed stochastic neighbor embedding (t‐SNE) [71] was performed. The analysis revealed three distinct clusters, representing different structural and electronic environments of zinc complexes (see Supporting Information: Figure S1). From each cluster, 15 molecules were randomly selected, yielding a final dataset of 45 representative complexes. The integration of SOAP descriptors with K‐means clustering facilitated comprehensive structural sampling while minimizing computational cost.

### 2.3 Quantum Chemical Calculations

All quantum‐chemical calculations were performed using Turbomole version 7.8 [72]. The geometries were optimized using DFT, with the r2SCAN‐3c [73] functional and the def2‐mTZVPP basis set. The r2SCAN‐3c functional was selected for geometry optimization based on its demonstrated accuracy in characterizing zinc(II) coordination compounds, as shown in recent benchmark studies [74]. NMR shielding tensors were calculated on the optimized structures using the GIAO method with the B3LYP [75, 76] functional at the def2‐TZVP level of theory. Solvent effects were incorporated using the implicit COSMO solvation model for five different solvents: Acetone (ε=20.7), chloroform (ε=4.8), dimethyl sulfoxide (DMSO, ε=46.7), methanol (MeOH, ε=32.7), and tetrahydrofuran (THF, ε=7.6). These solvents were chosen because they are typical for studies involving metal complexes. The calculated isotropic magnetic shielding values (σ) were converted to chemical shifts (δ) relative to tetramethylsilane (TMS) using the relationship:

(2) δ=σ(TMS)−σ.

The reference shielding value, σ(TMS), was calculated at the same level of theory in each respective solvent environment. This conversion from absolute shielding constants to relative chemical shifts facilitates direct comparison with experimental NMR data [77].

### 2.4 Machine Learning

Multiple regression models were evaluated to predict NMR chemical shifts based on the structural features encoded by SOAP descriptors. The SOAP vectors were generated using a cutoff radius of 6 Å, eight radial basis functions, and an angular momentum expansion up to order six, as implemented in the DScribe package. The ML models were trained using geometries optimized at the GFN2‐xTB level of theory, while the target NMR chemical shift values were obtained from higher‐level DFT calculations. To account for solvent effects, the dielectric constants (ε) of the respective solvents were included as explicit numerical features alongside the SOAP descriptors. We systematically compared eight machine learning algorithms: Random forest [78], gradient boosting regression [79], kernel ridge regression [80], support vector regression [81], gaussian process regression [82], XGBoost [83], lightGBM [84], and decision tree [85]. These algorithms were selected to represent diverse learning approaches, including ensemble methods (random forest, gradient boosting regression, XGBoost, lightGBM), kernel‐based methods (kernel ridge regression, gaussian process regression), and simpler decision tree models, enabling comprehensive benchmarking of prediction capabilities. The ML workflow was implemented using the scikit‐learn Python library (version 1.5.1) [86], with XGBoost and lightGBM implemented through their respective Python packages.

The dataset was split at the molecule level into 80% training (180 complex‐solvent combinations, 4060 H atoms) and 20% test (45 structures, 935 H atoms), ensuring all hydrogen atoms from a given complex remained in the same set. This prevents data leakage and provides a reliable assessment of model generalization. A comparison of performance differences between atom‐level and molecule‐level splitting strategies is provided in Supporting Information: Section S2.1, Table S3. To ensure a fair and consistent comparison across models, a fixed train‐test split was applied throughout the analysis. The hyperparameters of the models were optimized using a five‐fold cross validation scheme on the training set. Finally, the model performance was evaluated using mean absolute error (MAE), root mean square error (RMSE), and coefficient of determination ($R^{2}$).

### 2.5 Experimental Validation

To assess the robustness of our trained ML models, the calculated chemical shift values were directly compared against experimentally determined 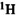 NMR shifts. A separate test set of 30 zinc(II) complexes and ligands not included in the training dataset was compiled, with experimental details and complete shift data available in the Supporting Information: Sections S3 and S4 and Figures S4–S50. For each structure, we generated three‐dimensional coordinates and optimized the geometries at the GFN2‐xTB level of theory, maintaining consistency with the computational approach used for our training structures. The SOAP descriptors were calculated for these optimized structures using identical parameters as in our model training phase. Using our trained ML models, we predicted the 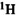 NMR chemical shifts across five solvent environments and compared them directly with experimental values obtained in the following solvents: Deuterated acetonitrile (ACN‐*d* 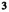, ε=37.5), deuterated chloroform (${\text{CDCl}}_{3},\varepsilon =4.8$), deuterium oxide (${\text{D}}_{2}\text{O},\varepsilon =78.5$), dimethyl sulfoxide (DMSO and DMSO‐*d* 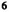, ε=46.7), and methanol (MeOH and MeOH‐$d_{4}$, ε=32.7). For dimethyl sulfoxide and methanol, both deuterated and non‐deuterated forms were used depending on the specific ligand or complex studied [87]. Despite their sensitivity to experimental conditions, exchangeable protons (NH, OH, COOH) were not excluded from the validation data which therefore provides a stringent real‐world test case.

## 3 Results and Discussion

### 3.1 Distribution of NMR Chemical Shifts in the Dataset

**FIGURE 2:**
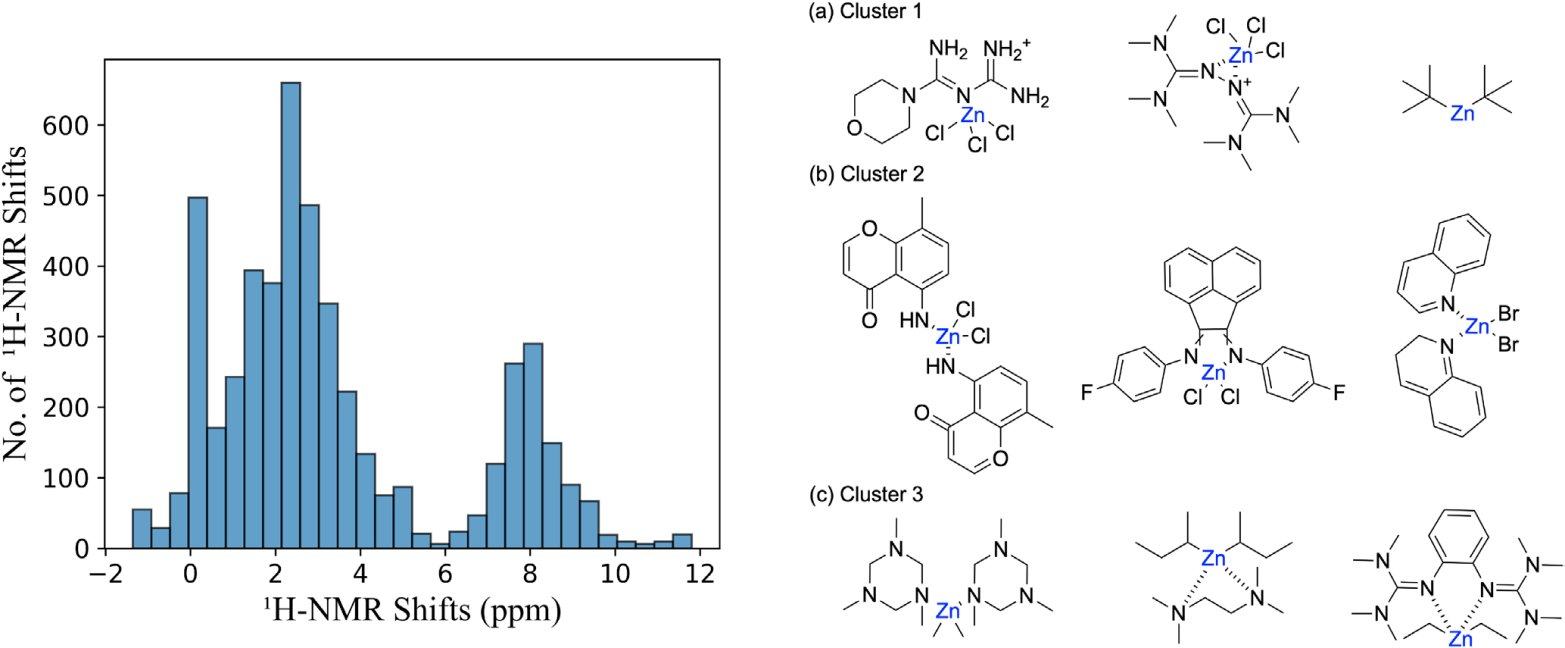
Overview of the dataset characteristics: (left) Distribution of calculated 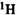 NMR chemical shifts in the dataset across five solvent environments. (right) Schematic representation of nine zinc complexes sampled from the three clusters, highlighting chemical diversity.

The complete dataset comprised 4995 hydrogen atoms across 45 zinc complexes in five solvents: Acetone, chloroform, DMSO, methanol, and THF. The shifts ranged from −1.37 to 11.80 ppm, with the majority of values concentrated between 1.42 and 4.53 ppm. The dataset exhibited a broad distribution, reflecting the diverse chemical environments of the protons in the selected zinc complexes (Figure 2, (left)).

To illustrate the variation within and across clusters, Figure 2 (right) shows a representative subset of nine complexes sampled from the three clusters. The selected zinc complexes exhibited varied coordination geometries (tetrahedral, octahedral configurations), different ligand types (featuring N‐, O‐ donor atoms), and diverse spatial arrangements. This structural diversity spanned from organometallic compounds containing zinc‐alkyl bonds (represented by di‐*sec*‐butyl and dimethyl derivatives) to complex coordination networks with heterocyclic ligands (such as quinoline and pyridyl‐thiazole systems). The varied structural configurations created unique electronic environments for hydrogen atoms throughout the ligand frameworks, which accounted for the wide distribution of chemical shifts observed in our NMR dataset.

To quantitatively assess the chemical diversity captured by our selection, we analyzed the coordination environments of the 45 complexes. The distribution included predominantly 4‐coordinate species (73.3%, tetrahedral), along with 6‐coordinate (11.1%, octahedral), 5‐coordinate (4.4%, trigonal bipyramidal), 3‐coordinate (4.4%), and 2‐coordinate (6.7%, organozinc compounds) geometries. Ligand donor analysis revealed N‐donor coordination (75.6%), mixed N,O‐coordination (17.8%), and other heteroatom donors including S and halides (6.7%).

Statistical validation comparing these 45 complexes to the full tmQM zinc database (5331 complexes) was performed using Kolmogorov‐Smirnov tests on SOAP descriptor principal components, showing no significant distributional differences for 9 out of 10 components (p>0.05), confirming representative sampling across the chemical space. Structural diversity analysis revealed that the selected complexes exhibit a diversity ratio of 1.23 relative to the full database, demonstrating that clustering‐based selection captured 23% greater structural variation than random sampling. Detailed statistical validation is provided in the Supporting Information: Section S1.5, Figures S2 and S3, and Tables S1 and S2.

### 3.2 Model Selection on the tmQM Dataset

We evaluated eight different machine learning models for their ability to predict proton NMR chemical shifts in zinc complexes across five different solvents, with performance metrics summarized in Table 1. All models were trained on the same SOAP descriptor features derived from the 45 representative zinc complexes, with solvent effects encoded via dielectric constants.

**TABLE 1:** Performance comparison of different ML models for predicting shifts, sorted by increasing MAE and decreasing $R^{2}$.

ML architecture	$R^{2}$ ^a^	MAE (ppm)^b^	RMSE (ppm)^c^	Training time (s)^d^
LightGBM	0.999	0.016	0.029	12.10
Random forest	0.999	0.021	0.033	11.50
Support vector regression	0.996	0.084	0.180	5.49
XGBoost	0.999	0.058	0.075	4.94
Gradient boosting regression	0.999	0.064	0.081	164.76
Gaussian process regression	0.959	0.365	0.617	176.19
Kernel ridge regression	0.885	0.747	1.028	1.15
Decision tree	0.879	0.758	1.059	0.18

^a^ Coefficient of determination ($R^{2}$).

^b^ Mean absolute error (MAE).

^c^ Root mean squared error (RMSE).

^d^ Training time in seconds.

Tree‐based ensemble methods demonstrated superior performance, with LightGBM achieving the highest accuracy (MAE = 0.016 ppm, RMSE = 0.029 ppm). This exceptional performance demonstrates the model's ability to accurately predict 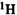 NMR shifts for unseen molecular structures, offering prediction times that are orders of magnitude faster than DFT‐based calculations while maintaining high accuracy. More significantly, while DFT calculations require several CPU‐hours per complex, our LightGBM approach trains in just 12.10 s, enabling high‐throughput screening of solvent effects across zinc complexes. Random forest performed comparably well (MAE = 0.021 ppm), while other tree‐based methods (XGBoost and Gradient boosting regression) revealed slightly higher errors but maintained excellent overall accuracy.

Notably, most models achieved remarkably high coefficient of determination values $\left(R^{2}>0.99\right)$, with only gaussian process regression, kernel ridge regression, and decision tree showing comparatively lower performance. The high $R^{2}$ values indicate the models' ability to account for nearly all variance in the chemical shift data, critical for accurate NMR prediction across a wide range of chemical environments.

**FIGURE 3:**
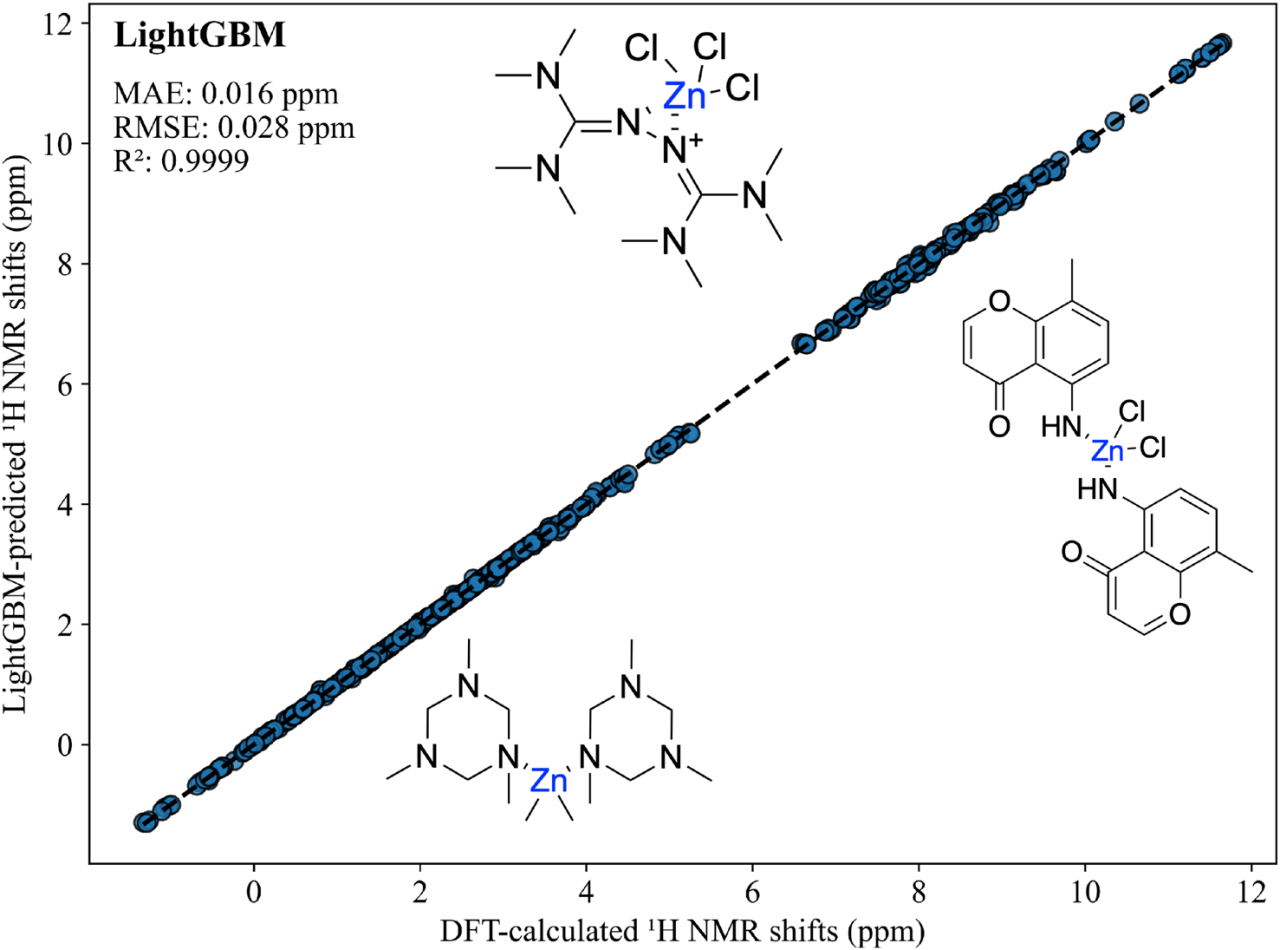
Correlation between LightGBM‐predicted and DFT‐calculated H NMR chemical shifts across five solvent environments. The schematic representation of the molecular structures are shown according to their corresponding chemical shift values.

Figure 3 presents a correlation plot between LightGBM‐predicted and DFT‐calculated 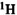 NMR shifts across all five solvents. The predicted values exhibit excellent agreement with DFT‐calculated shifts across the entire observed range (−1.37 to 11.80 ppm), demonstrating the model's consistent performance across diverse proton environments.

The superior performance of tree‐based methods reflects their ability to capture the non‐linear relationships between local chemical environments and resulting NMR shifts, consistent with other ML‐based NMR prediction studies across diverse chemical systems [33, 35, 88]. This further supports that SOAP descriptors effectively encode the local atomic environments governing NMR chemical shifts, as demonstrated in prior studies applying SOAP‐based machine learning models to diverse molecular and solid‐state systems [38, 89, 90].

### 3.3 Experimental Validation

Based on the performance metrics, we selected LightGBM as our best‐performing model for experimental validation and further analysis. To evaluate the robustness of our LightGBM model beyond computational datasets, we validated its predictions against experimentally measured 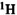 NMR shifts for a diverse set of zinc complexes and ligands with 654 hydrogen environments, not included in the training dataset [87]. These complexes are based on various binding motifs, including nitrogen donors, oxygen donors, and mixed N,O‐coordination systems. An overview of these different binding motifs can be found in the Supporting Information: Figure S2.

**FIGURE 4:**
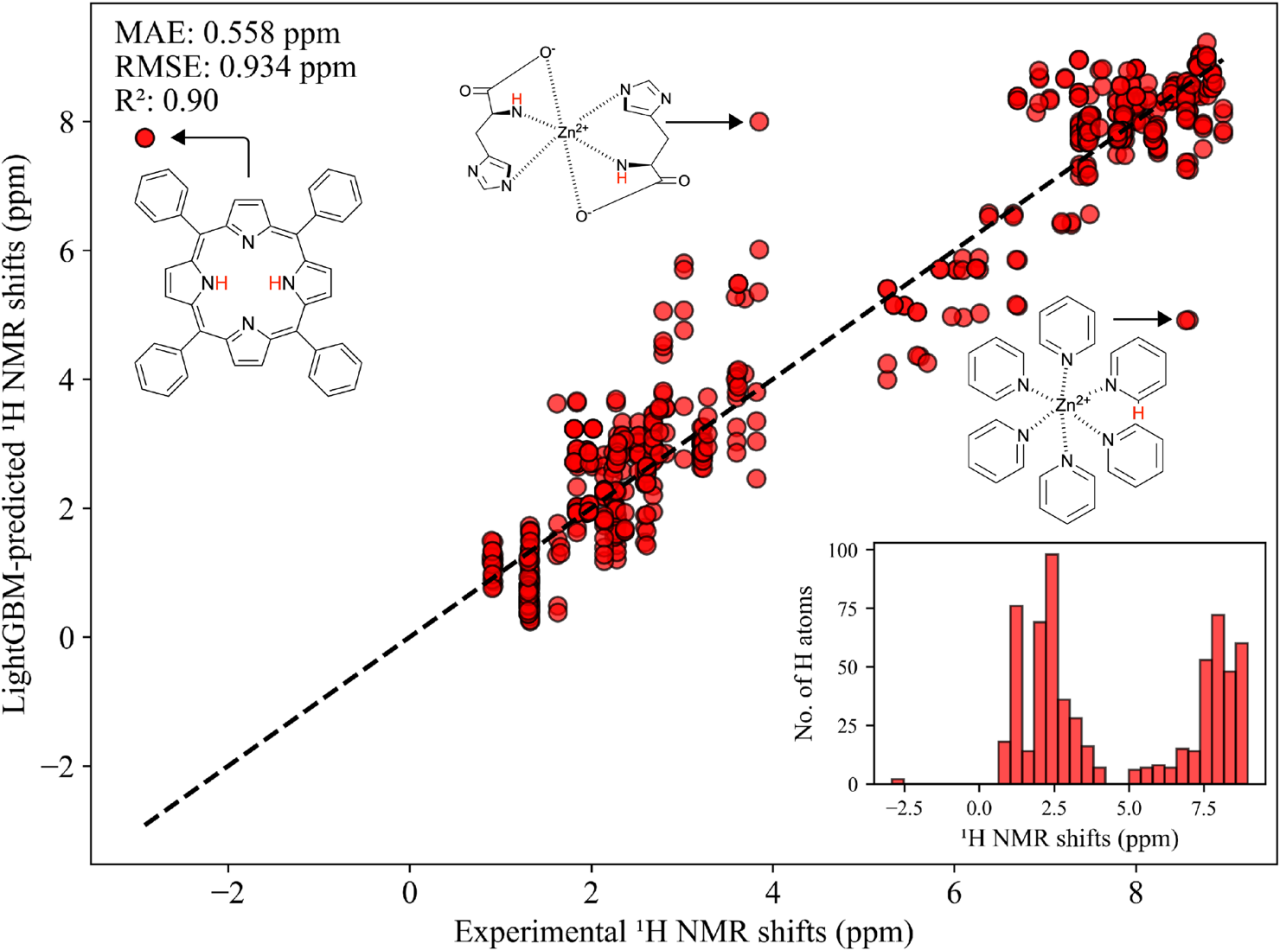
Predicted vs. experimental 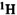 NMR shifts across five solvents using the LightGBM model. The model achieved $R^{2}=0.90$, MAE = 0.56 ppm, and RMSE = 0.93 ppm. Outlier complexes are indicated. The inset shows the distribution of experimental 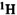 NMR chemical shifts in the test dataset.

Figure 4 presents a parity plot comparing predicted and experimental chemical shifts across the five studied solvents. Overall, the model achieved good agreement with experimental data, yielding an $R^{2}$ of 0.90, MAE of 0.56 ppm, and RMSE of 0.93 ppm. This indicates reasonable consistency between predicted results and experimentally measured values, effectively capturing the trend of 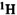 NMR shifts.

**FIGURE 5:**
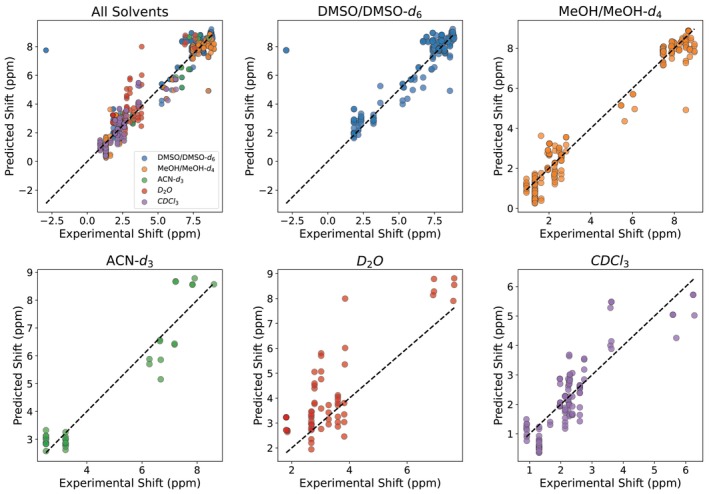
Multi‐panel correlation analysis between experimental and predicted 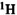 NMR shifts using the LightGBM model. The top left panel shows the aggregate performance across all solvent environments, while the remaining panels present solvent–specific correlations for DMSO/DMSO‐$d_{6}$, MeOH/MeOH‐$d_{4}$, ACN‐$d_{3}$, ${\text{D}}_{2}\text{O}$ and ${\text{CDCl}}_{3}$.

To assess our model's transferability across individual solvent environments, we performed a systematic solvent‐dependent analysis of the prediction accuracy. As presented in Figure 5 and Table 2, the model performed exceptionally well for MeOH/MeOH‐*d* 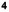 ($R^{2}=0.957$) and ACN‐$d_{3}$ ($R^{2}=0.913$), indicating strong correlation between ML predicted and experimental values for both solvents. In DMSO/DMSO‐$d_{6}$ and ${\text{CDCl}}_{3}$, the model maintained good performance with $R^{2}$ values of 0.79 and 0.63, respectively. However, predictions for ${\text{D}}_{2}\text{O}$ revealed considerably weaker correlation ($R^{2}=0.27$). Notably, neither ${\text{D}}_{2}\text{O}$ nor ACN‐$d_{3}$ were included in our original training set, making the strong performance in ACN‐$d_{3}$ ($R^{2}=0.913$) particularly impressive and demonstrating the model's ability to extrapolate to new solvent environments based on dielectric properties alone.

**TABLE 2:** Performance evaluation of the LightGBM‐predictions for experimental NMR shifts across different solvents, sorted by increasing MAE and decreasing $R^{2}$.

Solvent	$R^{2}$	MAE (ppm)	RMSE (ppm)
MeOH and MeOH‐$d_{4}$	0.957	0.484	0.646
ACN‐$d_{3}$	0.913	0.513	0.628
DMSO and DMSO‐$d_{6}$	0.786	0.544	1.179
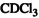	0.632	0.534	0.663
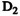 O	0.269	0.928	1.217

The weaker performance in ${\text{D}}_{2}\text{O}$ likely reflects the limitations of representing solvent effects solely through dielectric constants, particularly for solvents with strong hydrogen‐bonding capabilities that introduce additional complexities not fully captured by this simplified representation [91]. The distinctive physicochemical properties of ${\text{D}}_{2}\text{O}$ create solute‐solvent interactions that significantly influence NMR shifts beyond what can be represented through implicit dielectric models [50, 92]. These findings align with previous studies highlighting the complexity of accurately modeling aqueous environments for spectroscopic predictions and suggest that incorporating explicit water molecules or specialized solvation models may be necessary for improving predictions in strongly protic solvents [50, 93, 94].

Overall, the LightGBM model demonstrated robust performance, with an average MAE of 0.56 ppm for 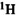 NMR shifts across all solvents. This accuracy is comparable to that typically achieved by DFT calculations [95] but at a fraction of the computational cost.

### 3.4 Analysis of Prediction Outliers

Among the experimental validation structures, we identified several outliers that presented significant challenges for the LightGBM model. While removing these hydrogen environments would decrease the prediction error and improve the overall performance, analyzing the outliers provides valuable insights into the fundamental limitations of our model and workflow.

**FIGURE 6:**
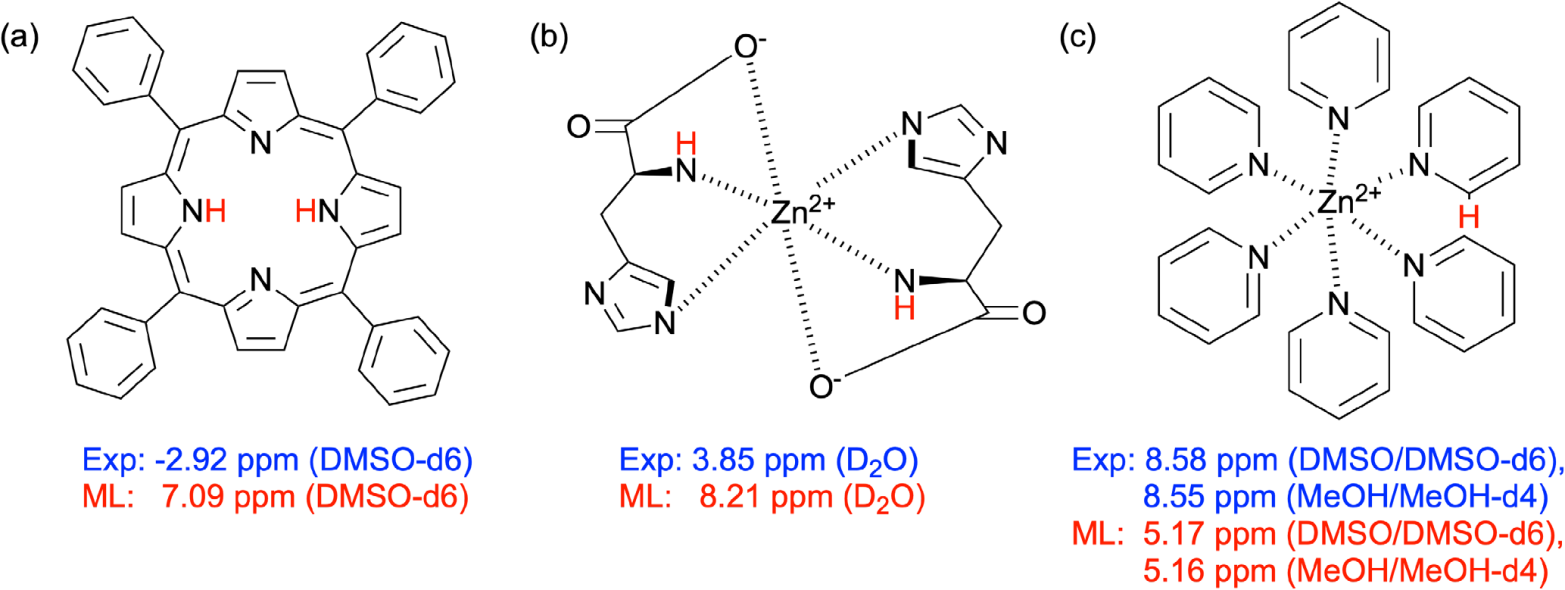
Schematic representation of the structural outliers exhibiting significant prediction errors: (a) Tetraphenylporphyrin ligand showing extreme upfield shift due to ring current effects; (b) *L*‐histidine zinc carbonate complex with challenging backbone amide prediction in ${\text{D}}_{2}$O; and (c) *hexakis*(pyridine)zinc(II) complex with prediction challenges for protons adjacent to coordinating nitrogen atoms. The experimental *vs.* LightGBM‐predicted 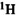 NMR shifts are shown for key proton environments.

A tetraphenylporphyrin (TPP) ligand (Figure 6a) exhibited an extremely shielded proton with an experimental chemical shift of −2.92 ppm. This extreme upfield shift falls well outside our training dataset range (−1.37 to 11.80 ppm), presenting a particularly challenging test case for model extrapolation. The LightGBM model predicted 7.09 ppm, significantly underestimating the upfield shift. This pronounced shift can be attributed to the strong ring‐current in TPP's aromatic system, which generates a powerful magnetic shielding for protons positioned above the macrocycle plane [96, 97]. Porphyrin structures generate considerably stronger ring current effects than typical coordination complexes, creating shielding environments inadequately represented in our training data. This discrepancy is consistent with previous studies showing that conventional ML descriptors often fail to fully capture the magnetic anisotropy effects in extended π‐systems [98].

Another notable outlier was observed in the *L*‐histidine zinc(II) carbonate complex in ${\text{D}}_{2}\text{O}$ (see Figure 6b), specifically with the backbone amide proton (N‐H). This backbone amide proton exhibited substantial prediction errors of 4.36 ppm compared to the experimental value. This observation reflects known challenges in accurately predicting NMR parameters for zinc‐bound histidine residues, where coordination can dramatically alter electronic environments around specific nuclei [99]. Moreover, zinc(II)‐histidine complexes can adopt multiple coordination geometries depending on experimental conditions, further complicating predictions [100]. Additionally, as discussed earlier, our representation of ${\text{D}}_{2}\text{O}$, as an implicit solvent, through dielectric constants alone fails to capture the complex hydrogen bonding network that significantly influences chemical shifts in aqueous environments.

In the *hexakis*(pyridine)zinc(II) complex ([Zn(py)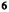]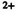) (see Figure 6c), protons adjacent to the coordinating nitrogen atoms also exhibited substantial prediction errors in both DMSO/DMSO‐$d_{6}$ and MeOH/MeOH‐$d_{4}$ solvents. This discrepancy likely stems from the complex electronic effects arising from metal‐ligand coordination in zinc(II) pyridine complexes. The sp^2^‐hybridized nitrogen lone pair in pyridine forms a σ‐bond with a vacant metal orbital, significantly altering the electronic environment of the pyridine ring [101]. Previous studies have demonstrated that these electronic perturbations particularly affect the protons proximal to the coordination site [102].

While these outliers exhibit substantial deviations, they constitute a small fraction of the validation set and arise from chemical features under‐represented in the training data: Charged species, macrocyclic ligands with pronounced ring current effects, and strongly hydrogen‐bonding solvents where implicit solvation proves inadequate. For the majority of neutral zinc (II) complexes, the model maintains practical accuracy (MAE = 0.56 ppm) suitable for structural characterization. When investigating systems containing these challenging structural motifs, complementing ML predictions with experimental measurements or targeted DFT calculations for critical assignments is advisable.

Despite these specific limitations, the overall performance of our LightGBM model across the majority of test compounds demonstrates its broad applicability for rapid and accurate prediction of solvent effects on 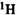 NMR shifts in zinc(II) complexes.

## 4 Conclusion

In summary, we introduce a machine learning framework for predicting 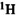 NMR chemical shifts in zinc(II) complexes and ligands across a wide range of solvent environments. We evaluated eight ML architectures trained on SOAP descriptors derived from 45 representative zinc (II) complexes selected via K‐means clustering from the tmQM database, with solvent effects implicitly encoded through dielectric constants. The dataset was split at the molecule level to ensure rigorous evaluation of generalization to unseen structures. Tree‐based ensemble methods exhibited superior performance, with LightGBM achieving exceptional accuracy (MAE = 0.016 ppm, RMSE = 0.028 ppm, $R^{2}=0.99$) on independent test complexes, reducing computation time from hours to seconds while maintaining high predictive accuracy. Upon experimental validation with an external dataset of 654 hydrogen atoms across 30 structures, the LightGBM model maintained robust performance ($R^{2}=0.90$, MAE = 0.56 ppm) across multiple solvents, showing particularly strong correlation in MeOH/MeOH‐$d_{4}$ ($R^{2}=0.96$), acetonitrile ($R^{2}=0.91$), and DMSO/DMSO‐$d_{6}$ ($R^{2}=0.79$). Notably, the model's strong performance in acetonitrile, despite its absence from the training dataset, demonstrates remarkable transferability, indicating the model's capacity to extrapolate to new solvent environments based on dielectric properties alone. Conversely, performance limitations observed in ${\text{D}}_{2}\text{O}$ suggest the need for improved descriptors that better account for hydrogen bonding in highly protic solvents. Furthermore, we identified structural motifs presenting significant predictive challenges, including macrocyclic ligands with strong ring currents, certain amino acid coordination environments, and charged complexes with heterocyclic ligands directly bound to metal centers. These findings suggest that supplementing SOAP descriptors with specialized features capturing electronic anisotropy and quantum mechanical descriptors of metal‐ligand interactions would further enhance model performance in these challenging chemical environments. Overall, our approach demonstrates that machine learning models, when trained on strategically selected structural descriptors and high‐quality quantum chemical data, can effectively bridge the gap between computational efficiency and accuracy in predicting solvent‐dependent NMR properties. This methodology offers a valuable tool for rapidly assessing spectroscopic properties of zinc (II) complexes across various solvent environments, with potential extensions to other transition metal systems.

## Funding

This work was supported by the “Deutsche Forschungsgemeinschaft” under the framework of the priority program SPP 2363 “Utilization and Development of Machine Learning for Molecular Applications–Molecular Machine Learning” (GR 4482/6; SCHU 1229/63‐1; project number 497115849). S.G. further highly acknowledges funding by the German Science Foundation via SFB/TRR 234 “CataLight,” project number 364549901, project C5. Acquisition of the 300 MHz Bruker Avance Neo NMR instrument was supported by grant 2022 FGI 0015 (Thüringer Aufbaubank TAB, European regional funds EFRE‐REACT).

## Conflicts of Interest

The authors declare no conflicts of interest.

## Supporting information




**Data S1:** Supporting Information.

## Data Availability

The data that support the findings of this study are openly available in Zenodo at https://doi.org/10.5281/zenodo.16966465, reference number 16966465. The repository includes raw Turbomole DFT outputs for 45 zinc complexes, solvent‐specific NMR calculations, experimental 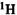 NMR spectra, compiled XYZ files for descriptor generation, and Jupyter notebooks implementing the clustering and LightGBM workflows used in this work.
