# Supplementary material for: Efficient Machine Learning Prediction of Solvent‐Dependent NMR Chemical Shifts in Zinc Complexes
Source: J Comput Chem. 2026 Apr 12;47(10):e70368. doi: 10.1002/jcc.70368 (PMC13071242; doi:10.1002/jcc.70368)
Supplement: Supplementary file 1 — Data S1: Supporting Information. [file JCC-47-0-s001.zip › 1HNMR_SI_rev.pdf]

Supporting Information

Efficient machine learning prediction of solvent-dependent ^{1}H NMR chemical shifts in zinc complexes

Jyothika R. Pillay,[1] Michael Ringleb,[2,3] Alexander Croy[1]*, Stefan Zechel,[2,3,4,5] Ulrich S. Schubert[2,3,4,5,6] and Stefanie Gräfe[1,7]

[1]Institute of Physical Chemistry (IPC), Friedrich Schiller University Jena, Helmholtzweg 4, 07743 Jena, Germany

[2]Institute for Organic and Macromolecular Chemistry (IOMC), Friedrich Schiller University Jena, Humboldtstr. 10, 07743 Jena, Germany

[3]Jena Center for Soft Matter (JCSM), Friedrich Schiller University Jena, Philosophenweg 7, 07743 Jena, Germany

[4]Helmholtz Institute for Polymers in Energy Applications (HIPOLE Jena), Lessingstrasse 12-14, 07743 Jena, Germany

[5]Helmholtz-Zentrum Berlin für Materialien und Energie (HZB), Hahn-Meitner-Platz 1, 14109 Berlin, Germany

[6]Center for Energy and Environmental Chemistry Jena (CEEC Jena), Friedrich Schiller University Jena, Philosophenweg 7a, 07743 Jena, Germany

[7]Fraunhofer Institute for Applied Optics and Precision Engineering (IOF), Albert-Einstein-Str. 7, 07745 Jena, Germany

*Email: alexander.croy@uni-jena.de

Table of Contents

1. **Selection of representative zinc complexes *via* clustering** 3
 1.1. **Description of structure selection** 3
 1.2. **CSD refcodes of selected complexes** 3
 1.3. **SOAP descriptor parameters** 4
 1.4. **K-means clustering parameters** 4
 1.5. **Statistical validation of representative selection** 4
  1.5.1. Principal component analysis 4
  1.5.2. Kolmogorov-Smirnov statistical tests 5
  1.5.3. Structural diversity analysis 6
2. **Machine learning predictions** 8
 2.1. **Comparison of atom-level *vs.* molecule-level splitting** 8
 2.2. **Machine learning hyperparameters** 8
  2.2.1. LightGBM 8
  2.2.2. Random forest 8
  2.2.3. Support vector regression 9
  2.2.4. XGBoost 9
  2.2.5. Gradient boosting regressor 9
  2.2.6. Gaussian process regressor 9
  2.2.7. Kernel ridge regressor 9
  2.2.8. Decision tree 9
3. **Experimental details** 10
 3.1. **Materials** 10
 3.2. **Instruments** 10
  3.2.1. Proton nuclear magnetic resonance (^{1}H NMR) spectroscopy 10
  3.2.2. Automated parallel synthesizer 11
 3.3. **Experiments** 11
  3.3.1. Preparation of zinc(II) (*bis* histidine) 11
  3.3.2. Preparation of zinc(II) ethylenediamine tetraacetic acid complex 11
  3.3.3. Preparation of *tris*-ethylenediamine zinc(II) 12
  3.3.4. Preparation of complexes and ligand solutions for the pyridine-based systems 12
  3.3.5. Preparation of complex and ligand solutions except pyridine-based systems 12
4. **^{1}H NMR spectra of the experimental investigated ligands and complexes** 14
5. **References** 37

1. Selection of representative zinc complexes *via* clustering

1.1. Description of structure selection

All the structures of the training dataset were obtained from the transition metal quantum mechanics (tmQM) dataset.[1] These structures were encoded using the smooth overlap of atomic positions (SOAP)[2] descriptors to capture their local atomic environments and geometric features. We applied k-means[3] clustering (k = 3) to this high-dimensional descriptor space to organize the structures into three broad structural categories. From each of the three clusters, we randomly selected 15 zinc complexes, resulting in a diverse subset of 45 structures for further analysis.

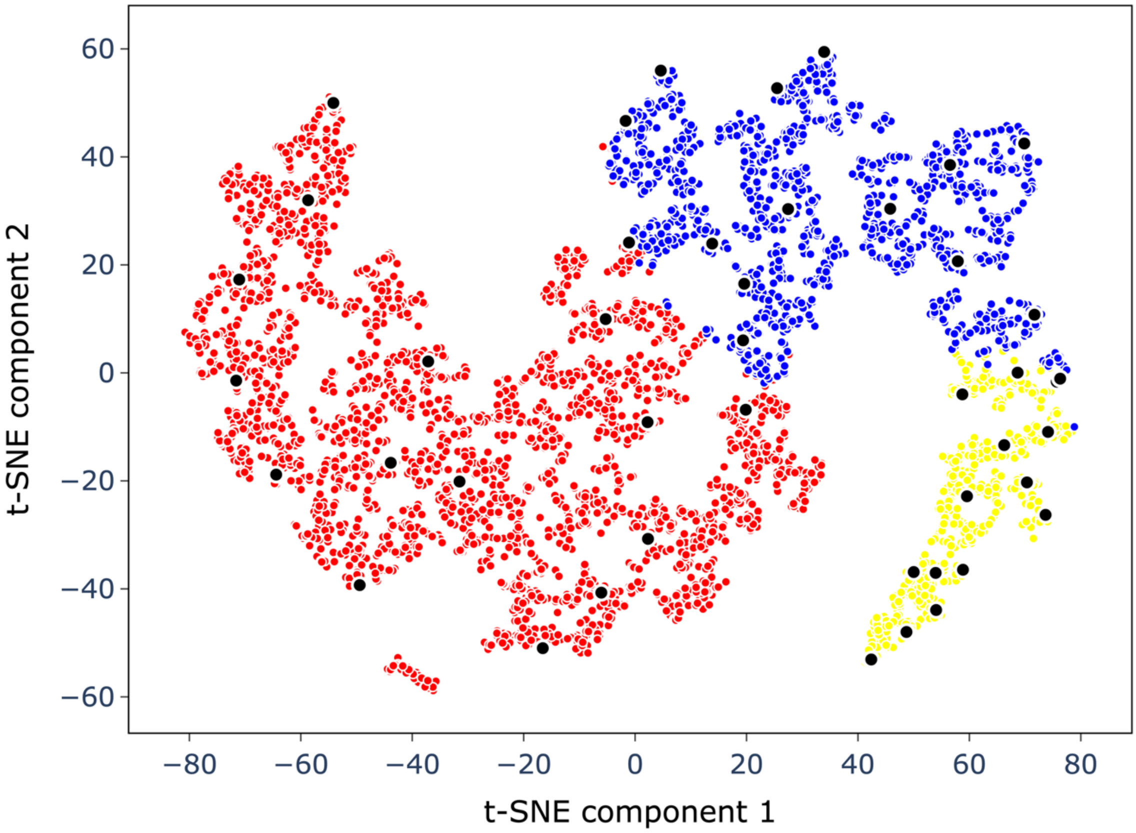

Figure S1: t-SNE visualization of 5,331 zinc complexes clustered based on SOAP descriptors. The 45 representative structures selected for the training dataset are highlighted with black markers.

1.2. CSD refcodes of selected complexes

The selected complexes correspond to the following cambridge structural database[4] (CSD) refcodes:

xebvoq, metvir, gosjih, rijhav, gevciv, supdiq, ohehit, jizcaw, erurit, tumqib, cudxuv, asecoq, nisvao, bekpuc, xisbad, hampan, forjus, qobtae, metxuf, govlei, govluy, qezlud, vulnat, iniczn, mimhul, litnil, nefvaw, onunan, nabviy, oqecod, notpiv, keqnus, unoqar, giylim, onohuv, vujjuh, eyetae, sajvab, wegquu, vuxtoz, cumbes, haydiw, yoqbai, eqaxur, ebegea.

1.3. SOAP descriptor parameters

The SOAP descriptors were generated using the DScribe[5] package with the following parameters:

- species_to_include = "H" (focus on hydrogen environments)
- rcut = 6.0 Å (cutoff radius)
- nmax = 8 (maximum number of radial basis functions)
- lmax = 6 (maximum degree of spherical harmonics)
- average = 'inner' (averaging method)
- periodic = false (non-periodic structures)
- sparse = false (dense representation)

1.4. K-means clustering parameters

K-means clustering was implemented using the scikit-learn[6] package with the following settings:

- n_clusters = 3
- n_init = 10 (number of initializations)

1.5. Statistical validation of representative selection

To validate that the 45 selected zinc complexes adequately represent the structural diversity of the full tmQM dataset (5,331 complexes), we performed comprehensive statistical analysis using SOAP descriptors computed for all hydrogen atoms in both datasets. This resulted in 252-dimensional SOAP feature vectors for each hydrogen atom. Principal component analysis (PCA) was performed on the standardized SOAP features to reduce dimensionality while preserving the major sources of structural variation. Kolmogorov-Smirnov (K-S) tests were then conducted on the first 10 principal components to statistically compare the distributions between the selected subset and the full dataset.

1.5.1. Principal component analysis

PCA reveals the main axes of structural variation in the zinc complex chemical space. The first two principal components (PC1 and PC2) capture 45.83% of the total variance (PC1: 27.81%, PC2: 18.02%), while the first 10 principal components collectively explain approximately 95% of the variance.

Table S1. Variance explained by principal components.

PC	Variance (%)	Cumulative (%)
PC1	27.81	27.81
PC2	18.02	45.83
PC3	13.09	58.92
PC4	9.37	68.29
PC5	8.99	77.28
PC6	8.78	86.06
PC7	4.55	90.61
PC8	2.52	93.13
PC9	1.06	94.19
PC10	0.88	95.07

1.5.2. Kolmogorov-Smirnov statistical tests

To quantitatively assess whether the 45 selected complexes are statistically representative of the full database, we performed two-sample K-S tests on the first 10 principal components. The K-S test evaluates the null hypothesis that two samples are drawn from the same distribution. A p-value > 0.05 indicates no significant difference between distributions, suggesting that the selected subset reflects the distribution of the complete dataset along the corresponding principal component.

Table S2. K-S test results comparing selected 45 complexes to full database.

Principal Component	KS Statistic	p-value	Pass (p>0.05)
PC1	0.2994	0.0005	No
PC2	0.1674	0.1458	Yes
PC3	0.1449	0.2778	Yes
PC4	0.1802	0.0969	Yes
PC5	0.1251	0.4512	Yes
PC6	0.1815	0.0930	Yes
PC7	0.0987	0.7400	Yes
PC8	0.0760	0.9414	Yes
PC9	0.1058	0.6608	Yes
PC10	0.1038	0.6829	Yes

9 out of 10 principal components show no significant distributional difference (p > 0.05) between the selected 45 complexes and the full database. Only PC1, which captures the largest variance in the dataset, shows a statistically significant difference. This is expected as the clustering-based selection intentionally samples from different regions of chemical space.

Figure S2 shows the distribution comparisons for the first 10 principal components. The overlapping distributions for PC2-PC10 ($p > 0.05$) confirm that our selection captures the structural diversity of the full database across multiple independent axes of variation.

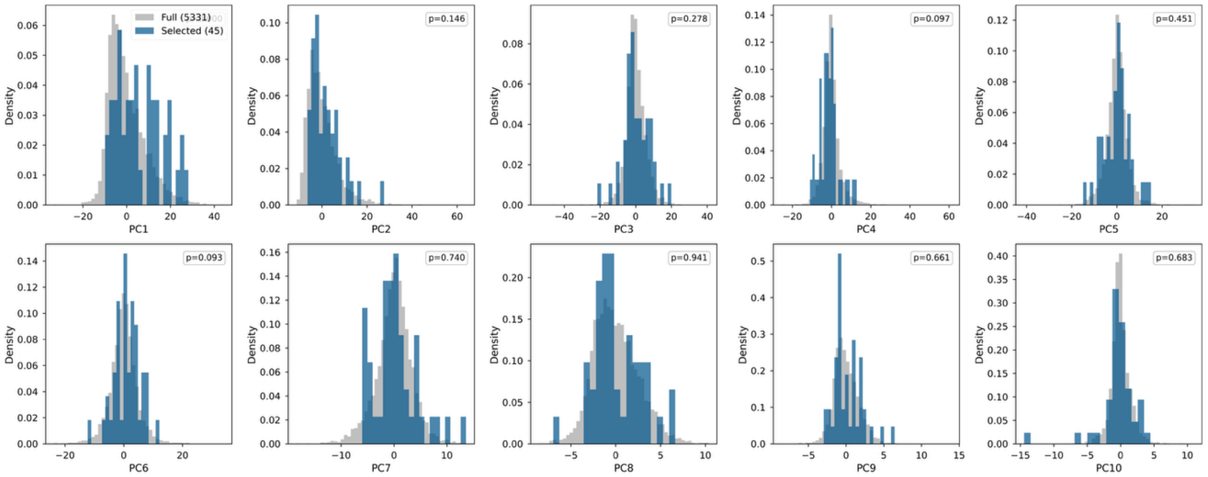

Figure S2. Distribution comparison between the full database and a selected subset for principal components PC1-PC10. Histograms show the distribution of PC scores for the full database (gray) and selected 45 complexes (blue). P-values from K-S tests are shown in each panel. Nine of ten components show no significant difference ($p > 0.05$), confirming representative sampling.

1.5.3. Structural diversity analysis

To further validate the representativeness of our selection, we computed pairwise Euclidean distances between SOAP descriptors in PC space (first 10 components). This provides a direct measure of structural diversity within each dataset.

Diversity metrics:

- Full dataset: Mean distance = 20.37, Std = 9.45
- Selected 45 complexes: Mean distance = 24.00, Std = 9.36
- **Coverage ratio: 1.23**

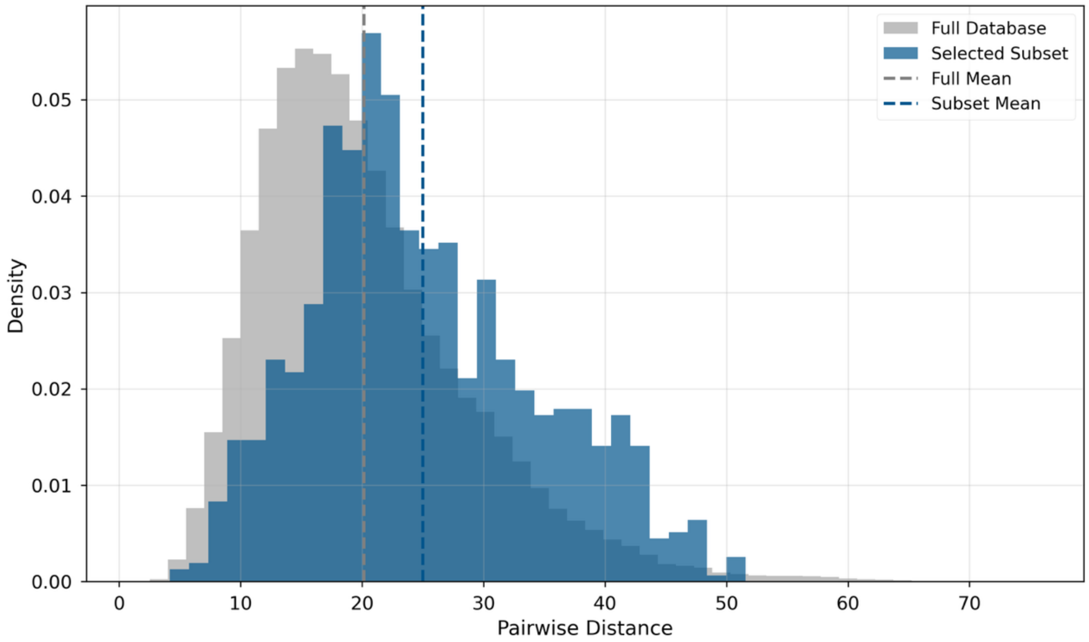

Figure S3: Structural diversity comparison. Distribution of the pairwise SOAP descriptor distances for a random sample from the full database (gray) and the 45 selected complexes (blue). The similar distribution widths confirm that the selected set captures the structural diversity of the full database.

The coverage ratio (mean distance in selected set / mean distance in full set) of 1.23 indicates that the selected complexes exhibit greater structural diversity than random sampling from the full dataset**.** This confirms that our K-means clustering approach successfully identified structurally diverse representatives that span the chemical space more effectively than random selection. The similar standard deviations (9.45 *vs.* 9.36) demonstrate that both datasets exhibit comparable variance in structural features.

Our statistical validation demonstrates that the 45 zinc complexes selected *via* K-means clustering on SOAP descriptors effectively represent the structural diversity of the full 5,331-complex tmQM dataset for neutral zinc complexes.

2. Machine learning predictions

2.1. Comparison of atom-level *vs.* molecule-level splitting

To evaluate model generalization, we compared two data splitting strategies:

Atom-level splitting: Individual hydrogen atoms randomly assigned to train/test sets (allows atoms from the same molecule in both sets)

Molecule-level splitting: All hydrogen atoms from a given complex remain together in either train or test set.

Table S3. Performance comparison of splitting strategies for LightGBM model.

Splitting Method	Train MAE (ppm)	Test MAE (ppm)	Test R^2
Atom-level	0.002	0.011	0.999
Molecule-level	0.028	0.016	0.999

The atom-level splitting shows notably lower training error (0.002 ppm), indicative of overfitting to the training set. In contrast, molecule-level splitting exhibits more balanced train-test performance (0.028 *vs.* 0.016 ppm), demonstrating that the model learns chemical relationships rather than memorizing specific molecular structures. The small difference in test MAE (0.005 ppm) between methods confirms robust model performance. Molecule-level splitting provides a more rigorous evaluation of generalization to truly unseen molecular structures and is therefore adopted throughout this work.

2.2. Machine learning hyperparameters

We evaluated eight different machine learning models for predicting ^{1}H NMR shifts. Each model was optimized through five-fold grid search cross validation. The final hyperparameters for each model are detailed below:

2.2.1. LightGBM

- num_leaves: 50
- learning_rate: 0.05
- n_estimators: 0
- subsample: 0.8
- colsample_bytree: 0.8

2.2.2. Random forest

- n_estimators: 1000
- max_depth: None
- max_features: ‘sqrt’
- min_samples_split: 2
- min_samples_leaf: 1
- bootstrap: False

2.2.3. Support vector regression

- kernel: 'rbf'
- C: 100
- gamma: 0.1
- epsilon: 0.01

2.2.4. XGBoost

- n_estimators: 100
- learning_rate: 0.05
- max_depth: 3
- min_child_weight: 1
- gamma: 0
- subsample: 0.8
- colsample_bytree: 0.8

2.2.5. Gradient boosting regressor

- n_estimators: 1000
- learning_rate: 0.05
- max_depth: 3
- n_iter_no_change: 10

2.2.6. Gaussian process regressor

- kernel: C(1.0, (1e-4, 1e1)) * RationalQuadratic(length_scale=1.0, alpha=1.0)

2.2.7. Kernel ridge regressor

- kernel: 'rbf'
- alpha: 1.0
- gamma: 0.1

2.2.8. Decision tree

- max_depth: 3
- min_samples_split: 2
- min_samples_leaf: 1
- splitter: ’best'

3. Experimental details

3.1. Materials

L-Histidine (> 99% purity), methacrylic acid (> 99%), acridine orange hemizinc salt, *bis*(2,4-pentanedionato)zinc(II) (> 96%), dichloro(*N,N,N',N'*-tetramethylethylenediamine) zinc(II) (> 98%), tetraphenylporphyrin (> 98%), 5,10,15,20-tetraphenyl-21H,23H-porphine zinc (> 98%), zinc(II) dodecanoate (> 98%), 2-(2-hydroxyphenyl) benzothiazole (> 98%) and *bis*[2-(2-benzothiazolyl)phenolato]zinc(II) (> 98%) were purchased from TCI.

2,2':6',2''-Terpyridine (> 98%), ethylenediamine tetraacetic acid disodium salt dihydrate (EDTA) (> 99%), ethylenediamine (> 98%), zinc(II) stearate, zinc(II) methacrylate, zinc(II) acetate dihydrate, acridine orange (base) (75% dye content), *N,N,N',N'*-tetramethylethylenediamine (> 99.5%) and dodecanoic acid (98%) were purchased from Sigma Aldrich.

Acetylacetone, zinc(II) carbonate and zinc(II) sulphate heptahydrate were used from old stocks, produced within the German democratic republic. Hence, a more precise annotation of the supplier is not possible.

Pyridine (> 99%), 2,2'-bipyridine (> 98%) and zinc(II) acetate (99.9%) were purchased from Thermo FisherScientific.

Dimethyl sulfoxide (99.7%) was purchased from Acros Organics.

Stearic acid (> 97%) was purchased from Merck-Schuchardt.

Glacial acetic acid (> 99.7%) was purchased from Fisher Scientific.

Methanol (99.9%) was purchased from VWR chemicals.

Tetramethylsilane (TMS) (99.9%) was purchased from abcr.

Ethanol was purchased from Brenntag in technical grade (99% purity).

Deuterated acetonitrile (ACN-*d3*), deuterated chloroform ($CDCl_3$), deuterated dimethyl sulfoxide (DMSO-*d6*), deuterated water (D_2O) and deuterated methanol (MeOH-*d4*) were purchased from Eurisotop.

3.2. Instruments

3.2.1. Proton nuclear magnetic resonance (^{1}H NMR) spectroscopy

^{1}H NMR spectra of the pyridine, bipyridine and terpyridine ligands and the corresponding complexes were recorded on a Bruker Avance Neo Nanobay (300 MHz) spectrometer equipped with a SampleJet sample changer and ^{1}H-probe at room temperature with 16 scans per spectrum if not otherwise stated. Chemical shifts (δ) are given in parts per million (ppm-scale) relative to the deuterated solvent.

^{1}H NMR spectra for the other complexes were either measured on a Bruker Avance NEO 300 (300.19 MHz, ^{1}H; 75.5 MHz, ^{13}C) with a 5 mm BBFO iProbe (with automatic sample loading system (SampleCasePlus) for high through put sample analysis) or on a Bruker Avance III (400.13 MHz, ^{1}H; 100.62 MHZ, ^{13}C) with a 5 mm PA BBO 400S1

BBF-H-D-05 Z probehead and a B-ACS 60 sample changer as indicated in the label of the figures. The spectra were measured at room temperature. Chemical shifts (δ) are given in parts per million (ppm-scale) relative to the deuterated solvent.

3.2.2. Automated parallel synthesizer

For the experiments connected to the pyridine-based ligands and complexes, an automated parallel synthesizer platform (Accelerator SLT 100), produced by the company Chemspeed was utilized. The platform consists of a liquid handling interface made up of a 4-needle-head which is connected *via* PTFE tubing to syringe pumps. The pumps are IMI Norgren V6 syringe drive pumps with either 1 or 10 mL fitting glass syringes, both provided by the company Chemspeed Technologies AG. The 4-NH can be taken up by an automated tool-exchange interface and transferred to each place inside the platform's footprint. The needles are then lowered to aspirate or dispense liquids in previously programmed zones. Further information is available in a previous publication.[7]

3.3. Experiments

A representation of all prepared complexes can be found in **Figure S4.**

3.3.1. Preparation of zinc(II) (*bis* histidine)

The histidine complex of zinc(II) was obtained utilizing a previously published procedure.[8]

256.15 mg (2.04 mmol) zinc(II) carbonate and 633.94 mg (4.08 mmol) *L*-histidine were dissolved in 40 mL deionized water in a 50 mL round bottom flask. The flask was thermostated in a preheated oil bath at 80 °C and the content was stirred for one hour. Afterwards, the water was removed under reduced pressure. A viscous substance remained in the flask. The flask was placed inside a drying oven at 40 °C for three days. After this period, a colorless solid formed inside the flask. 0.57 g of this colorless solid were obtained.

The ^{1}H NMR spectrum of the complex with annotated integrals is available in **Figure S34**.

3.3.2. Preparation of zinc(II) ethylenediamine tetraacetic acid complex

The tetraacetic acid complex of zinc(II) was synthesized adapting a published procedure.[9]

3.50 g (15.95 mmol) zinc(II) acetate dihydrate and 2.97 g (7.97 mmol) ethylenediamine tetraacetic acid disodium salt dihydrate (EDTA) were dispersed in 15 mL of deionized water in a 50 mL round bottom flask with a reflux condenser and a stirring bar. The flask was thermostated in a preheated oil bath at 165 °C while stirring. The solution was refluxed for one hour. Afterwards, the flask was taken out of the oil bath and cooled to room temperature. The precipitate inside the flask was filtered off, utilizing a fluted filter and afterwards washed with ca. 5 mL of deionized water. Subsequently, the precipitate was transferred to a petri dish and dried at room temperature for one day. Then, the petri dish was placed in a drying oven at 40 °C and the precipitate was dried for three days. 1.902 g of a colorless solid were obtained.

The ^{1}H NMR spectrum of the complex with annotated integrals is available in **Figure S36**.

3.3.3. Preparation of *tris*-ethylenediamine zinc(II)

The zinc(II) complex of ethylenediamine (EDA) was prepared according to literature.[10]

4.35 mL of a 0.20 M zinc(II) sulphate solution in deionized water were prepared from zinc(II) sulphate heptahydrate. 4.35 mL of a 0.61 M ethylene diamine solution in deionized water were prepared. Afterwards, the EDA solution was added dropwise to the stirred zinc(II) solution at room temperature. In the beginning of the addition, the mixture became turbid but cleared up again with the addition of more EDA solution. After the addition was finished, the solution was stirred for one hour at room temperature. Subsequently, 2.5 mL of ethanol were added and the complex precipitated as a colorless solid. The solution was transferred to a drying oven and dried for three days at 45 °C. 0.326 g of a colorless solid were obtained.

The ^{1}H NMR spectrum of the complex with annotated integrals is available in **Figure S38**.

3.3.4. Preparation of complexes and ligand solutions for the pyridine-based systems

The samples from the pyridine-based systems were prepared semi-automatically utilizing a previously published workflow.[7]

In short: A solution of zinc(II) acetate in a mixture of 10 v% deuterated solvent (DMSO-*d6* or MeOH-*d4*) with 90 v% HPLC-grade solvent (DMSO or methanol) was prepared. For the DMSO solvent mixture, as well, TMS was added for chemical shift calibration. Furthermore, solutions of pyridine, 2,2'-bipyridine or 2,2':6',2''-terpyridine, respectively, in the same solvent were prepared. Utilizing an automated parallel synthesizer, different volumes of the salt solutions were mixed with the pyridine-based solutions and samples were taken for NMR measurement. The spectra in the (**Figure S5** to **Figure S14**) represent either the first spectrum (pure ligand) or the spectrum of the mixture with a corresponding ratio of ligand to metal ion of 2:1 (for 2,2':6',2''-terpyridine), 3:1 (for 2,2'-bipyridine) or 6:1 (for pyridine) which corresponds to the expected ratios in the complex. Due to low solubility in the respective solvent mixtures, the complexes of zinc(II) acetate with 2,2'-bipyridine in methanol and the complex of zinc(II) acetate with 2,2':6',2''-terpyridine in DMSO could not be measured.

3.3.5. Preparation of complex and ligand solutions except pyridine-based systems

The solutions for the NMR investigation of the complexes and ligands were prepared by transferring the complex or ligand into a 1.5 mL Eppendorf-vial. Subsequently, 600 µL of the respective NMR solvent (ACN-*d3*, $CDCl_3$, DMSO-*d6*, D_2O or MeOH-*d4*) was added to the vial. The vial was closed and shaken. If solid was still visible inside

the vial, the supernatant was transferred to an NMR tube. Else, the whole solution was transferred to the NMR tube. Subsequently, the solutions were measured.

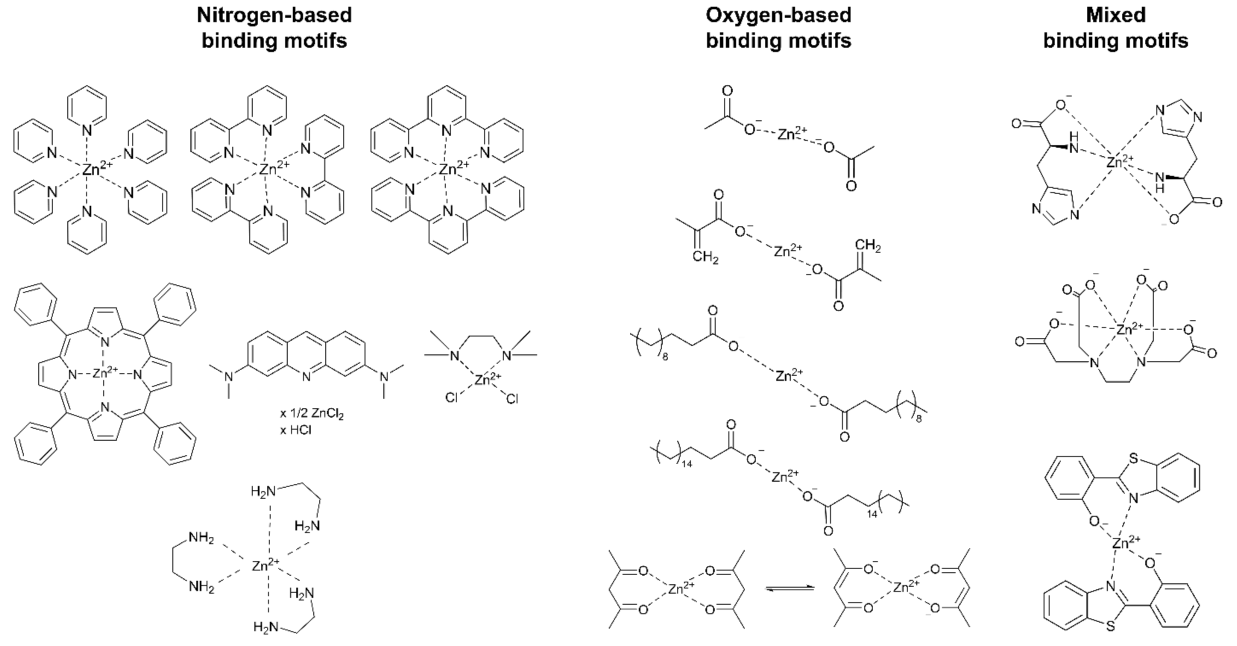

Figure S4: Schematic representation of the structure of the complexes which were experimentally prepared sorted by their binding motifs.

4. ^{1}H NMR spectra of the experimental investigated ligands and complexes

4.1. Spectra of pyridine-based ligands and complexes

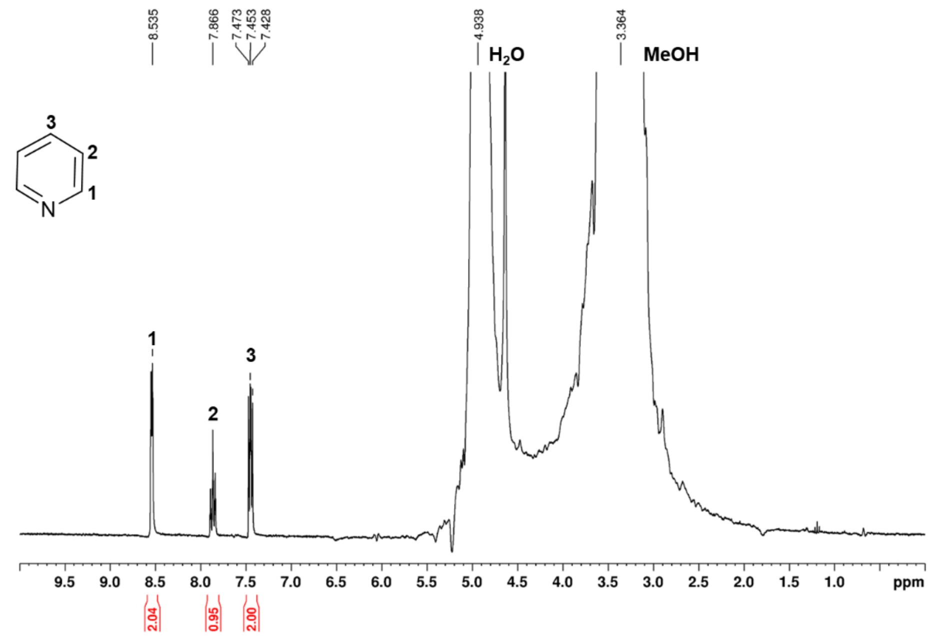

Figure S5: ^{1}H NMR spectrum of pyridine in a mixture of deuterated methanol (10 v%) and HPLC-grade methanol (90 v%) with peak assignments (300 MHz). Proton signal assignment based on peak integral values and literature reports.[11]

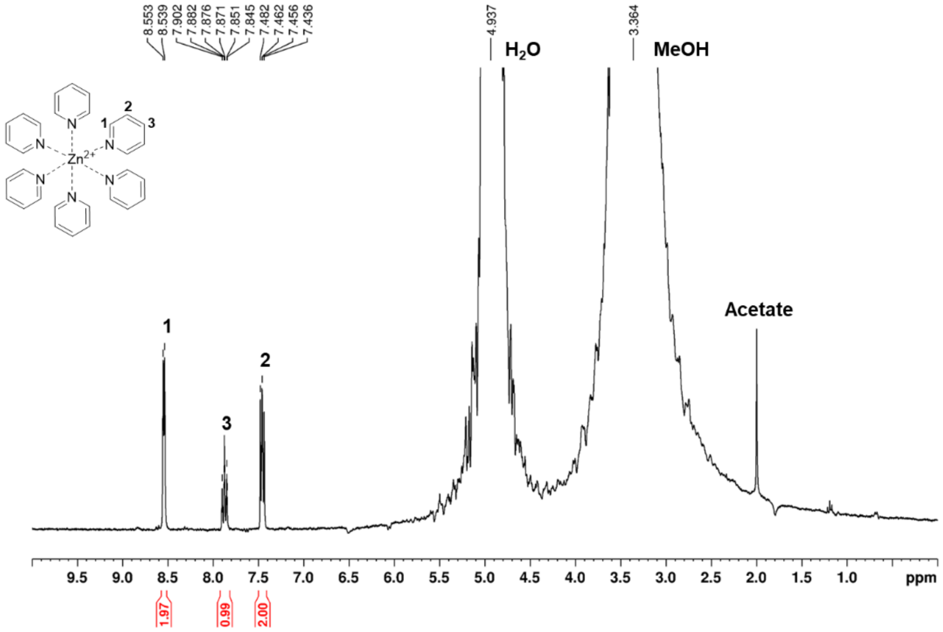

Figure S6: ^{1}H NMR spectrum of a complex of pyridine with zinc(II) acetate in a mixture of deuterated methanol (10 v%) and HPLC-grade methanol (90 v%) with peak assignments (300 MHz). Proton signal assignment based on peak integral values and literature reports.[11]

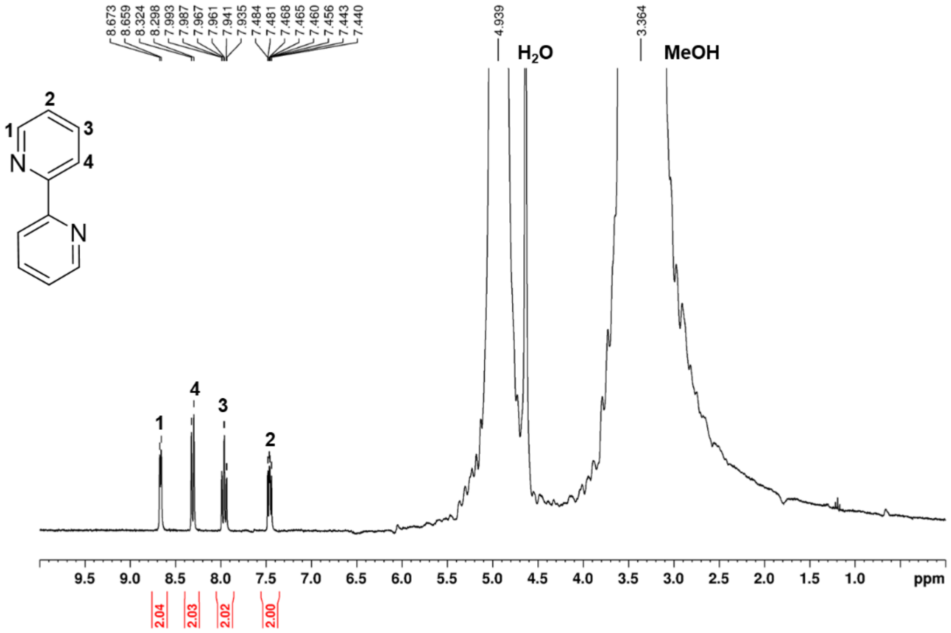

Figure S7: ^1H NMR spectrum of 2,2'-bipyridine in a mixture of deuterated methanol (10 v%) and HPLC-grade methanol (90 v%) with peak assignments (300 MHz). Proton signal assignment based on literature reports.[12]

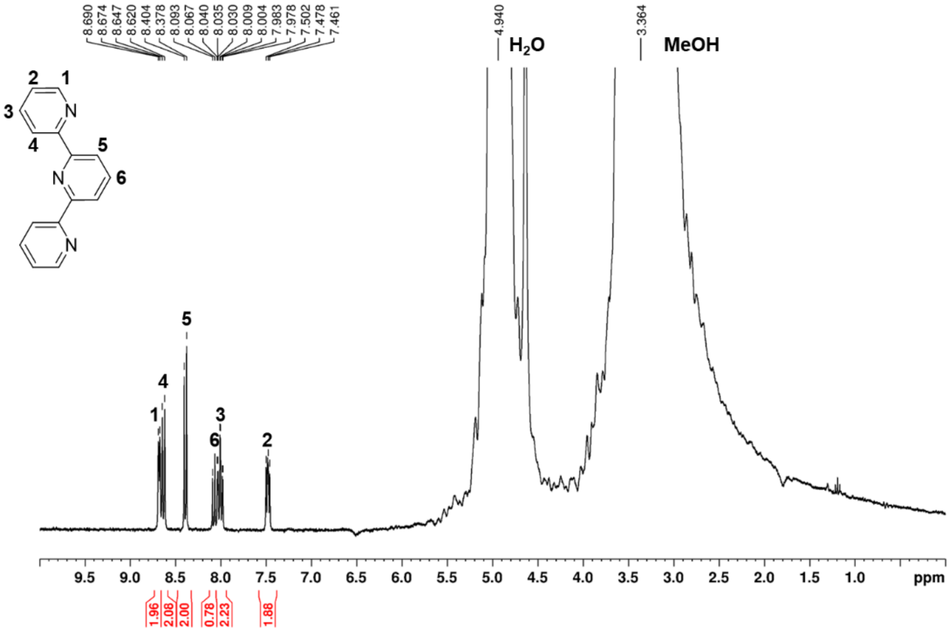

Figure S8: ^1H NMR spectrum of 2,2':6',2''-terpyridine in a mixture of deuterated methanol (10 v%) and HPLC-grade methanol (90 v%) with peak assignments (300 MHz). Proton signal assignment based on literature reports.[13]

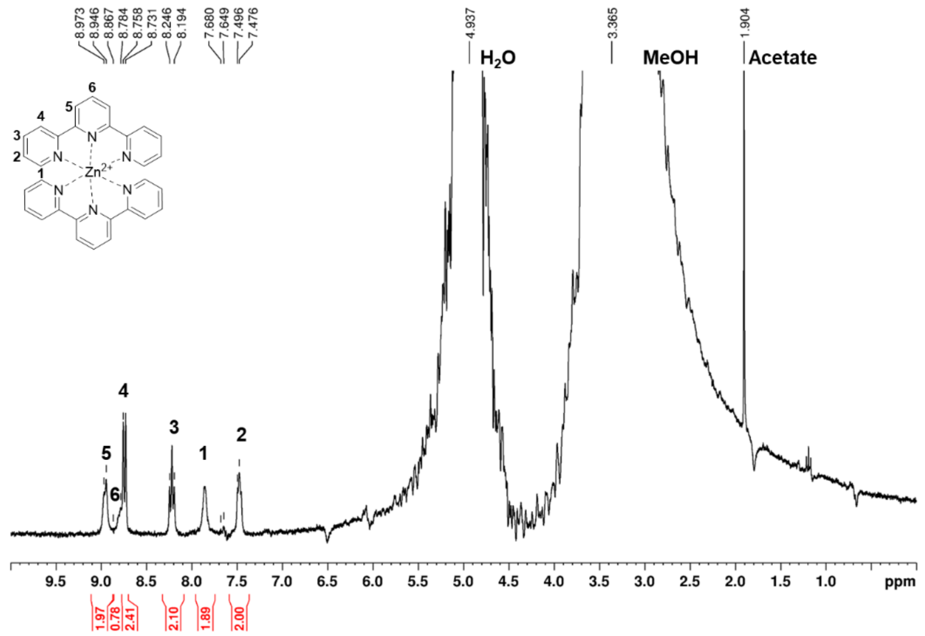

Figure S9: ^{1}H NMR spectrum of a complex of 2,2':6',2''-terpyridine with zinc(II) acetate in a mixture of deuterated methanol (10 v%) and HPLC-grade methanol (90 v%) with peak assignments (300 MHz). Proton signal assignment based on literature reports.[13]

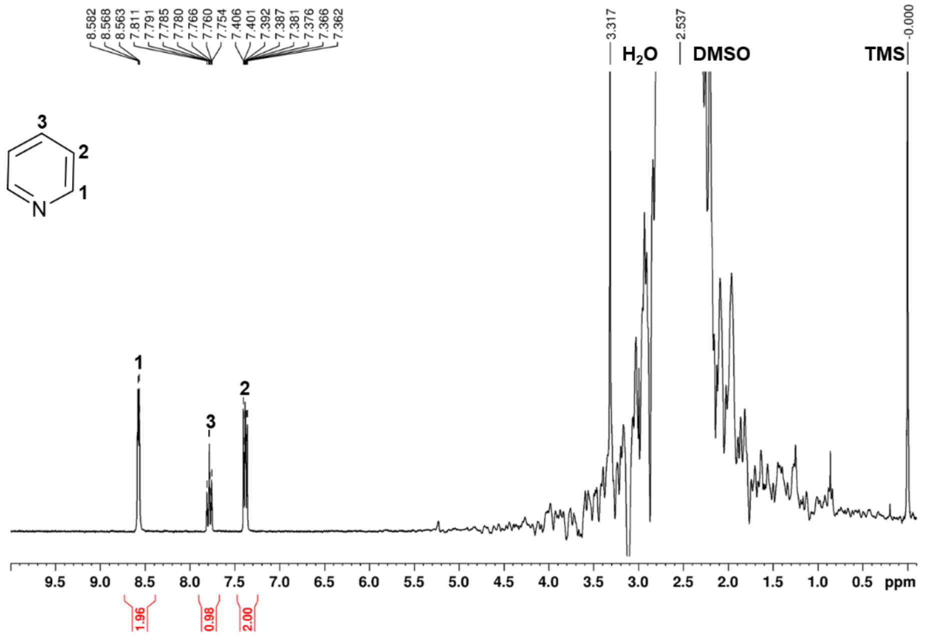

Figure S10: ^{1}H NMR spectrum of pyridine in a mixture of deuterated dimethyl sulfoxide (DMSO) (10 v%) and HPLC-grade DMSO (90 v%) and added TMS with peak assignments (300 MHz). Proton signal assignment based on peak integral values and literature reports.[11]

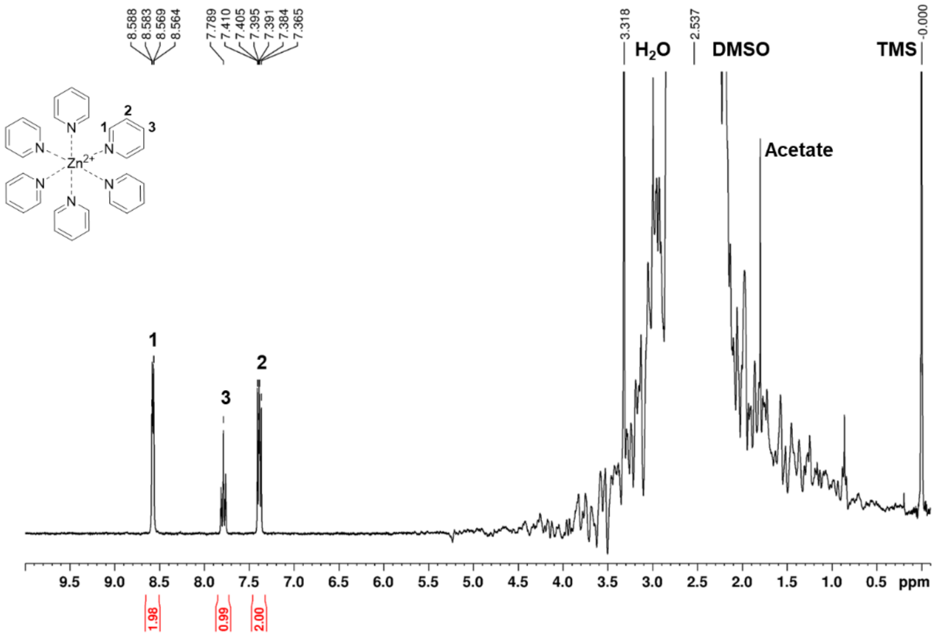

Figure S11: ^{1}H NMR spectrum of a complex of pyridine with zinc(II) acetate in a mixture of deuterated DMSO (10 v%) and HPLC-grade DMSO (90 v%) and added TMS with peak assignments (300 MHz). Proton signal assignment based on peak integral values and literature reports.[11]

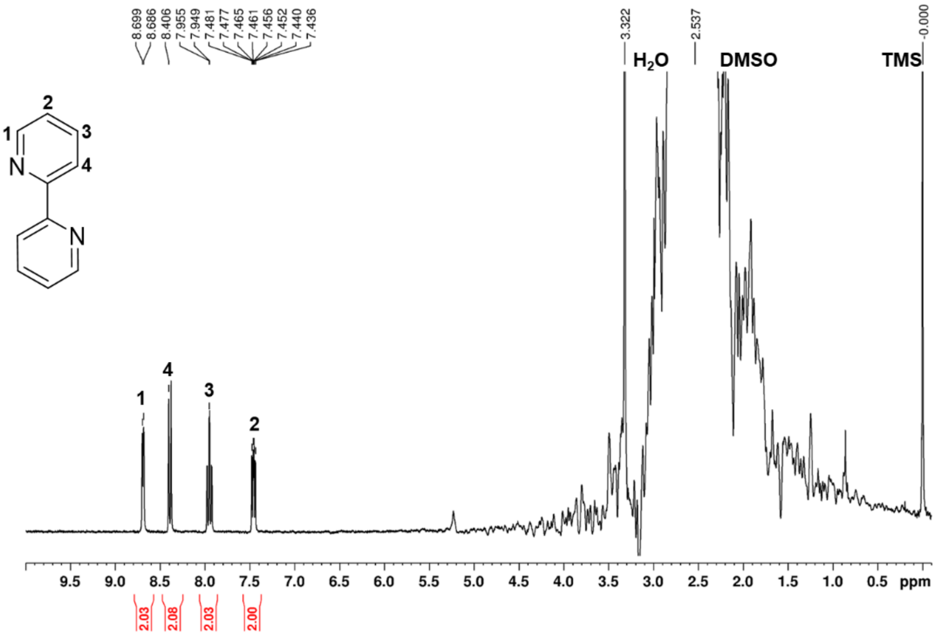

Figure S12: ^{1}H NMR spectrum of 2,2'-bipyridine in a mixture of deuterated DMSO (10 v%) and HPLC-grade DMSO (90 v%) and added TMS with peaks assignment (300 MHz). Proton signal assignment based on literature reports.[12]

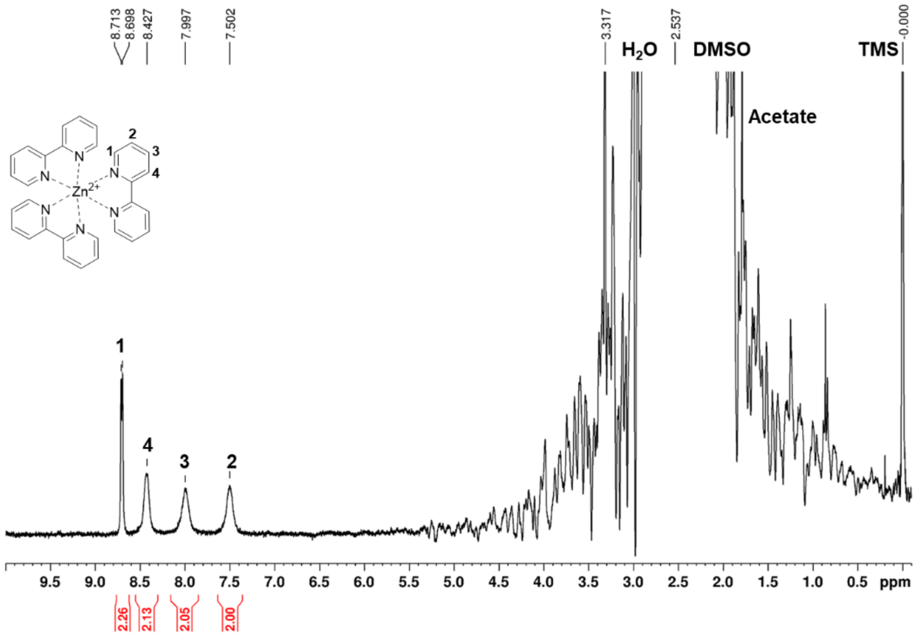

Figure S13: ^{1}H NMR spectrum of a complex of 2,2'-bipyridine with zinc(II) acetate in a mixture of deuterated DMSO (10 v%) and HPLC-grade DMSO (90 v%) and added TMS with peak assignments (300 MHz). Proton signal assignment based on literature reports.[14]

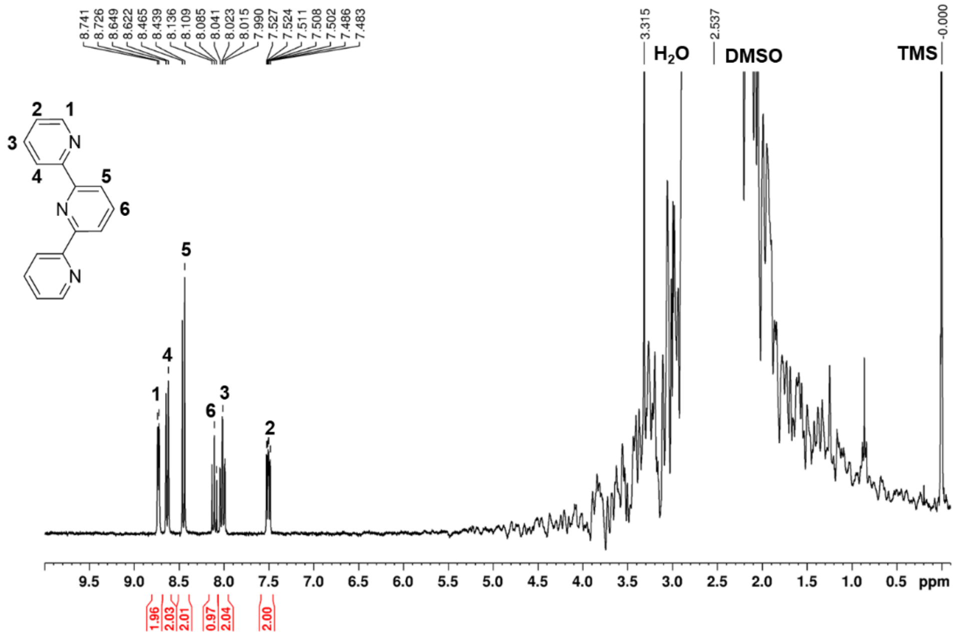

Figure S14: ^{1}H NMR spectrum of 2,2':6',2''-terpyridine in a mixture of deuterated DMSO (10 v%) and HPLC-grade DMSO (90 v%) and added TMS with peak assignments (300 MHz). Proton signal assignment based on literature reports.[12]

4.2. ^{1}H NMR spectra with peak assignments for further ligands and complexes

In the following all spectra for the non-pyridine-based systems are represented. All peaks, which are related to the ligand are annotated. However, acid and hydroxyl protons were not annotated in the structures shown in the spectra as they are generally not visible after complexation due to deprotonation.

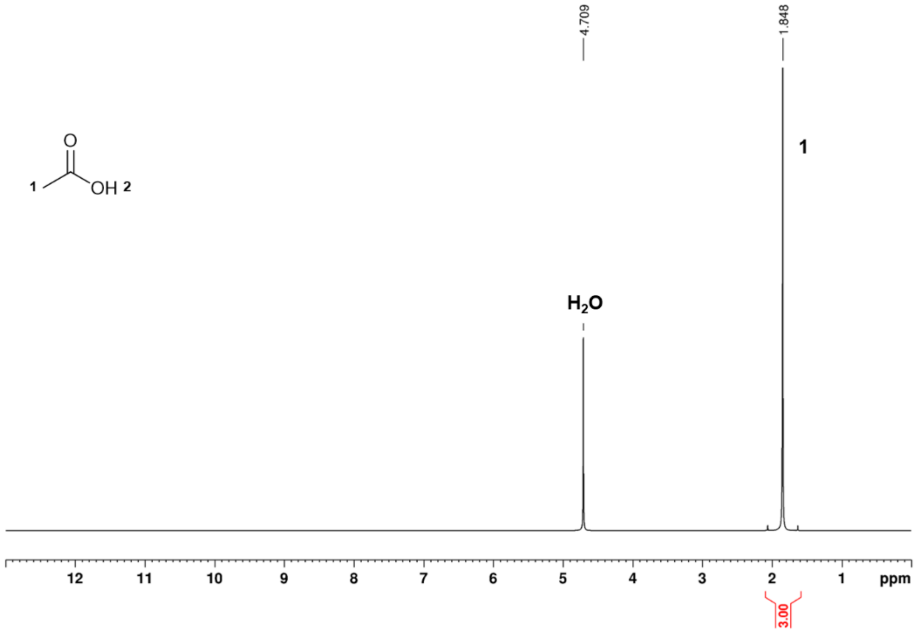

Figure S15: ^{1}H NMR spectrum of acetic acid with signal assignments (300 MHz, D_2O).

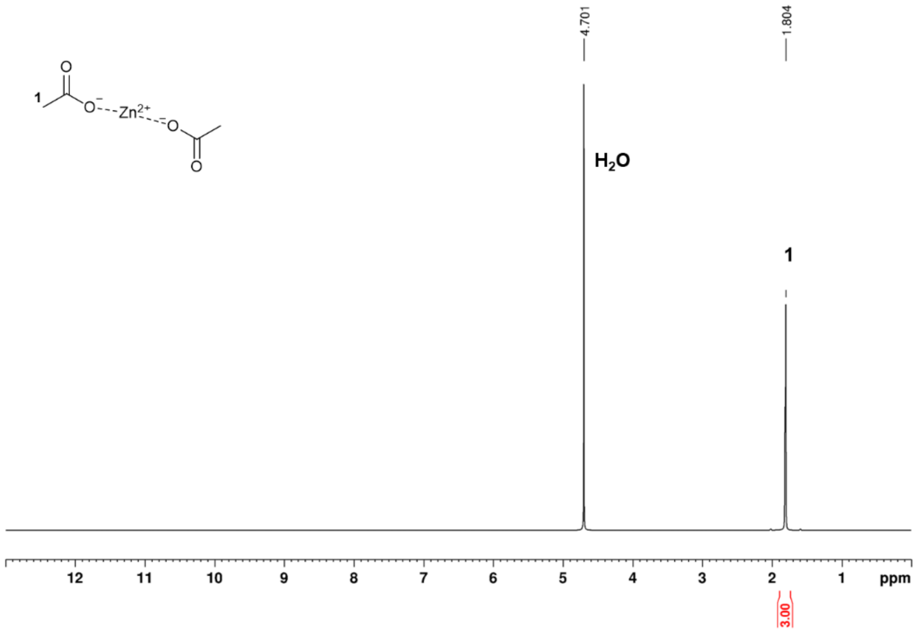

Figure S16: ^{1}H NMR spectrum of zinc(II) acetate dihydrate with signal assignments (300 MHz, D_2O).

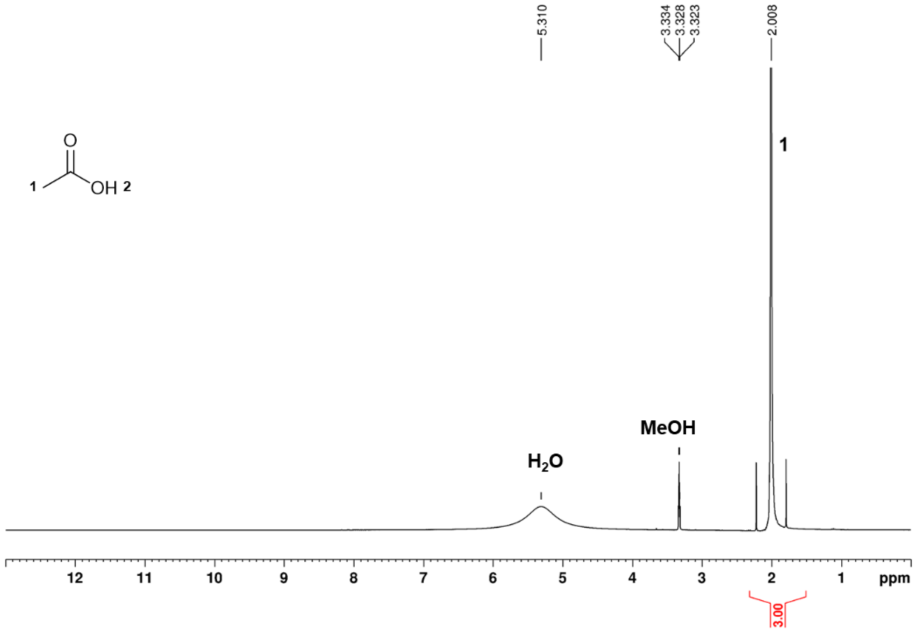

Figure S17: ^{1}H NMR spectrum of acetic acid with signal assignments (300 MHz, MeOH-*d4*).

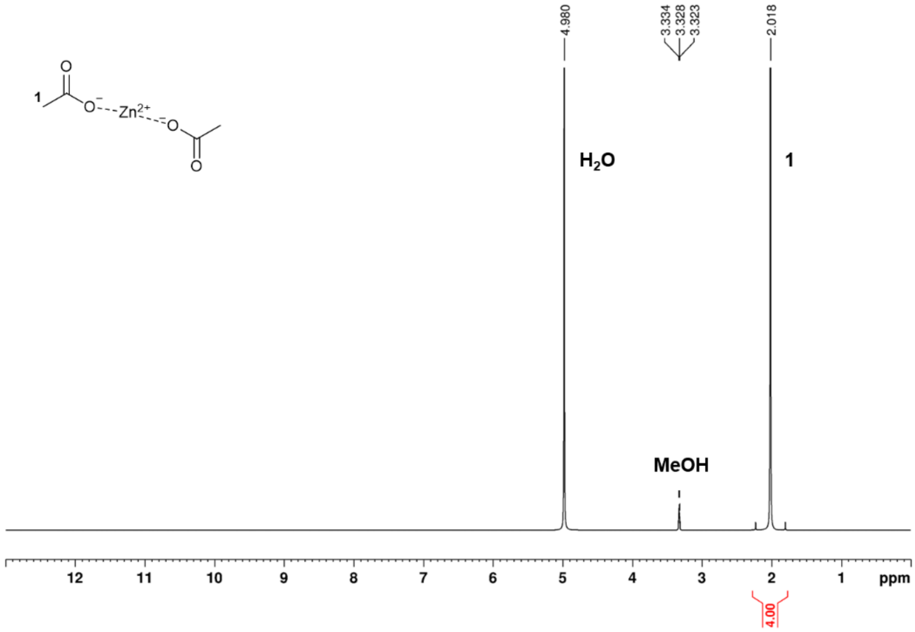

Figure S18: ^{1}H NMR spectrum of zinc(II) acetate dihydrate with signal assignments (300 MHz, MeOH-*d4*).

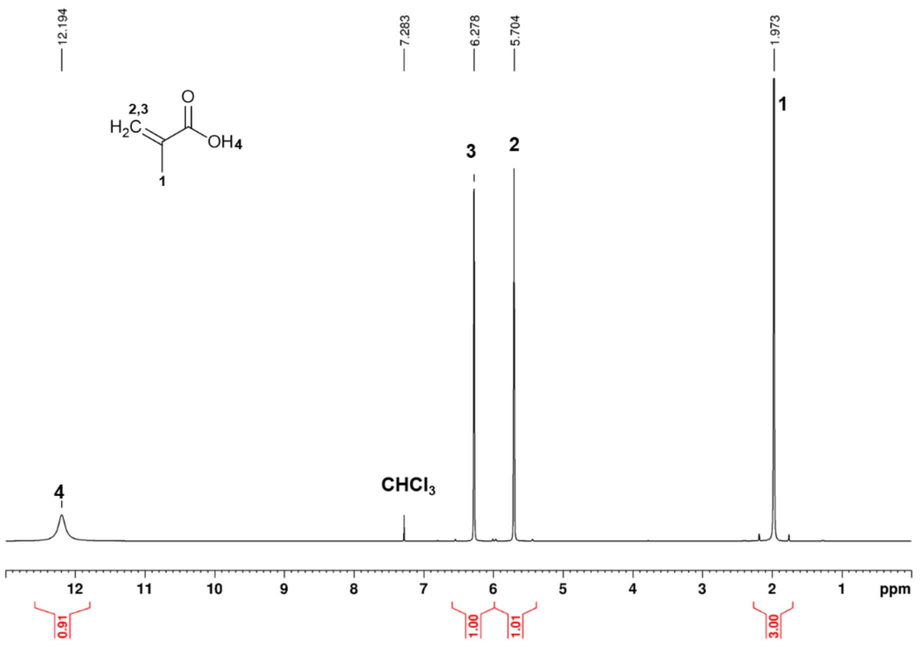

Figure S19: ^{1}H NMR spectrum of methacrylic acid with signal assignments (300 MHz, $CDCl_3$). Proton signal assignment based on literature reports.[15]

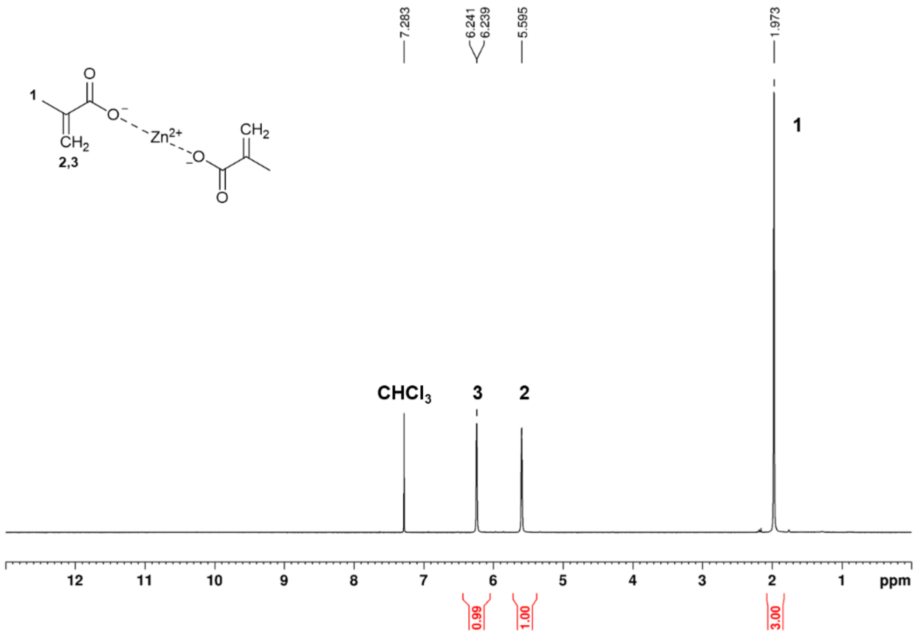

Figure S20: ^{1}H NMR spectrum of a zinc(II) methacrylate with signal assignments (300 MHz, $CDCl_3$). Proton signal assignment based on literature reports.[15]

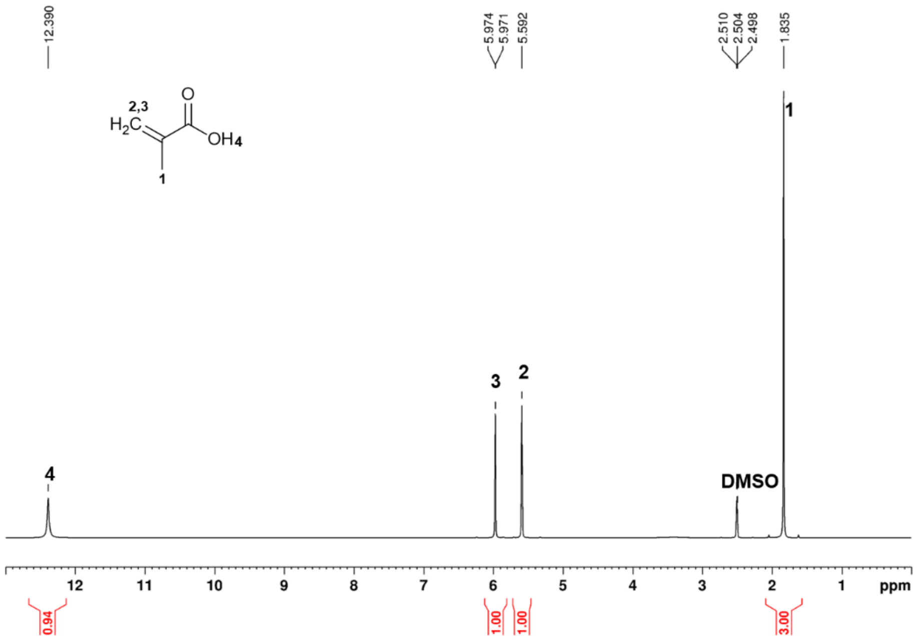

Figure S21: ^{1}H NMR spectrum of methacrylic acid with signal assignments (300 MHz, DMSO-*d6*). Proton signal assignment based on literature reports.[15]

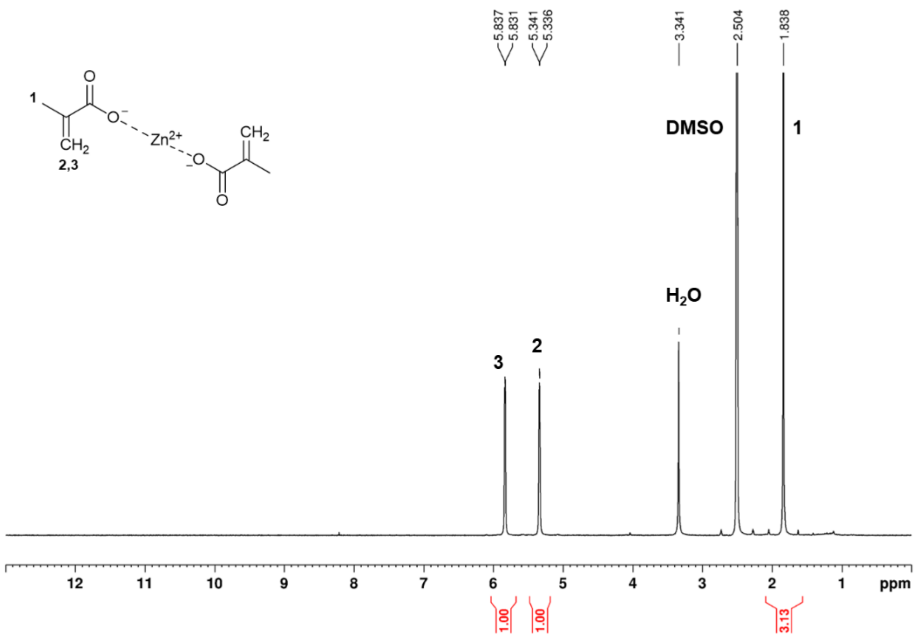

Figure S22: ^{1}H NMR spectrum of zinc(II) methacrylate with signal assignments (300 MHz, DMSO-*d6*). Proton signal assignment based on literature reports.[15]

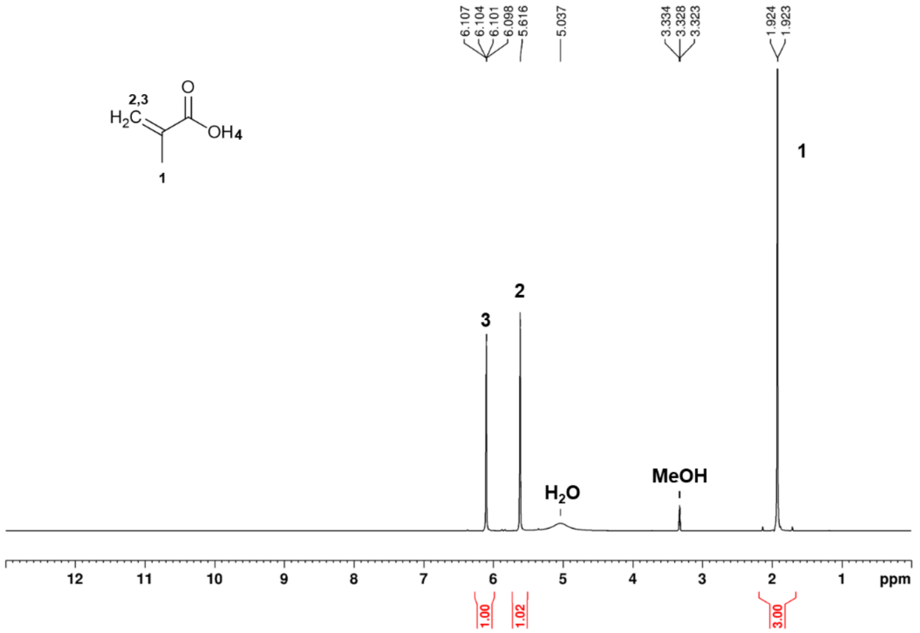

Figure S23: ^1H NMR spectrum of methacrylic acid with signal assignments (300 MHz, MeOH-*d4*). Proton signal assignment based on literature reports.[15]

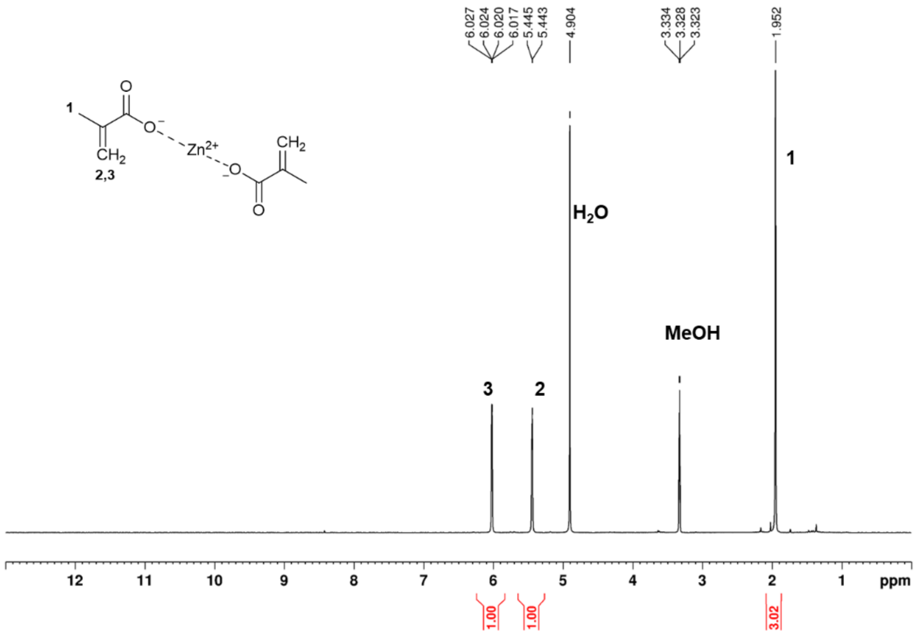

Figure S24: ^1H NMR spectrum of a zinc(II) methacrylate with signal assignments (300 MHz, MeOH-*d4*). Proton signal assignment based on literature reports.[15]

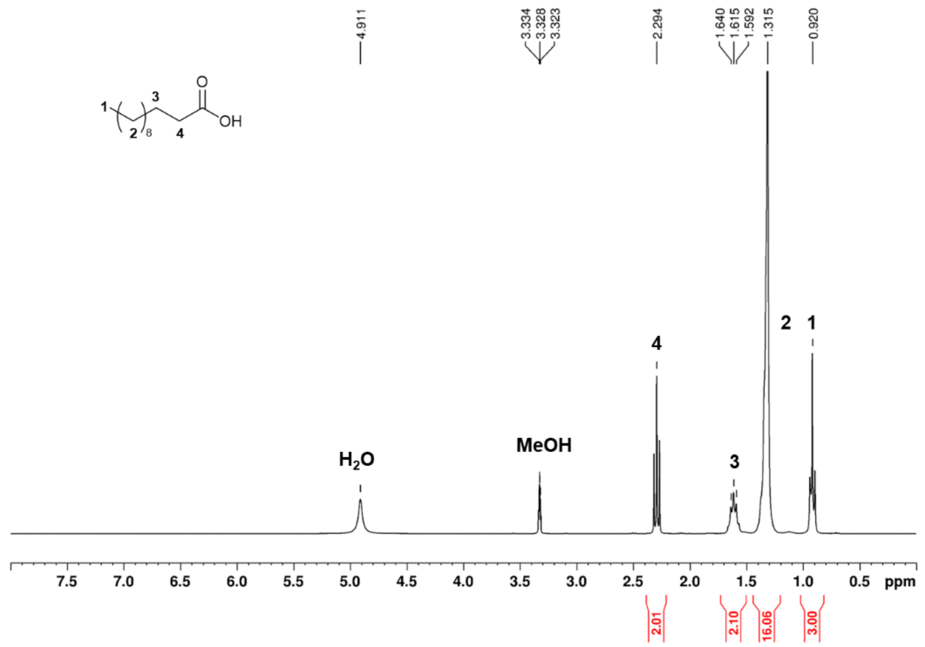

Figure S25: ^{1}H NMR spectrum of dodecanoic acid with signal assignments (300 MHz, MeOH-*d4*). Proton signal assignment based on literature reports.[16]

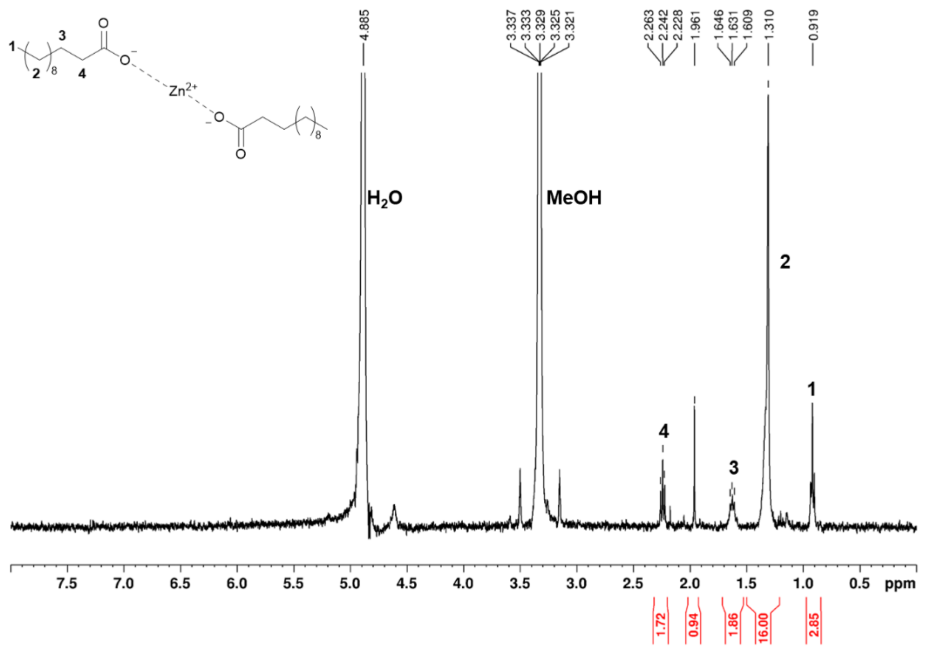

Figure S26: ^{1}H NMR spectrum of zinc(II) dodecanoate with signal assignments (400 MHz, MeOH-*d4*). Proton signal assignment based on literature reports.[16]

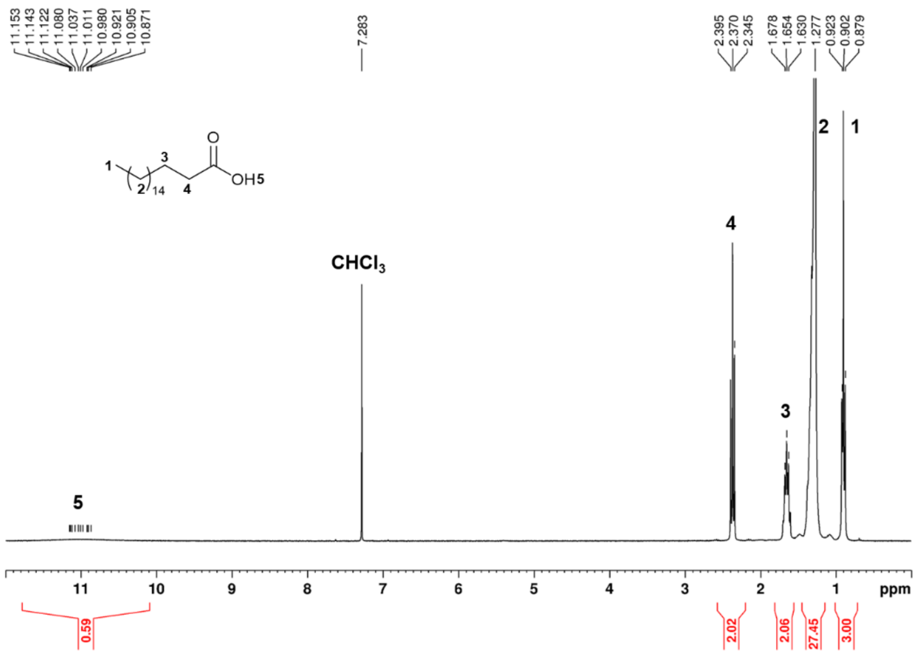

Figure S27: ^{1}H NMR spectrum of stearic acid with signal assignments (300 MHz, $CDCl_3$). Proton signal assignment based on literature reports.[17]

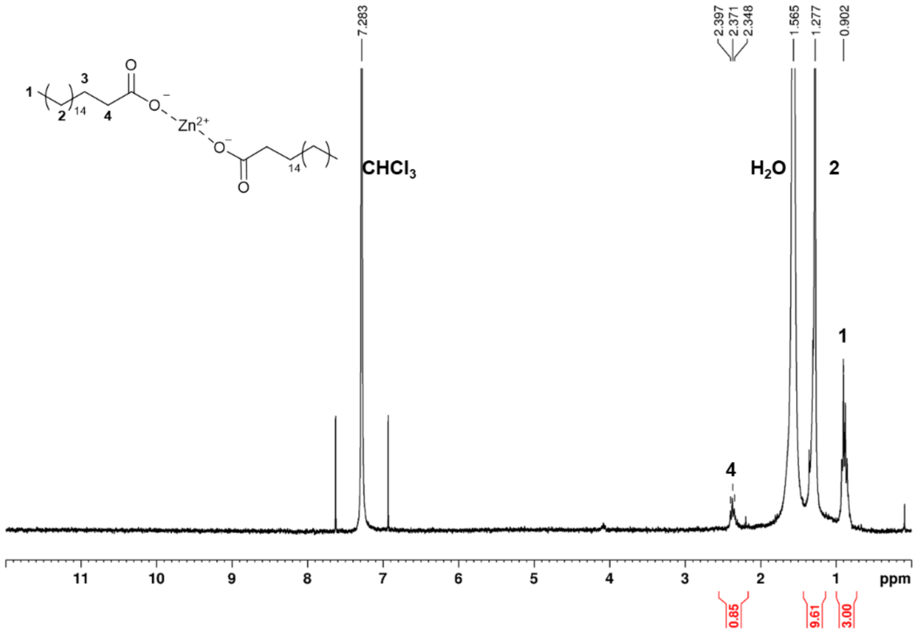

Figure S28: ^{1}H NMR spectrum of zinc(II) stearate with signal assignments (300 MHz, $CDCl_3$). Proton signal assignment based on literature reports.[17]

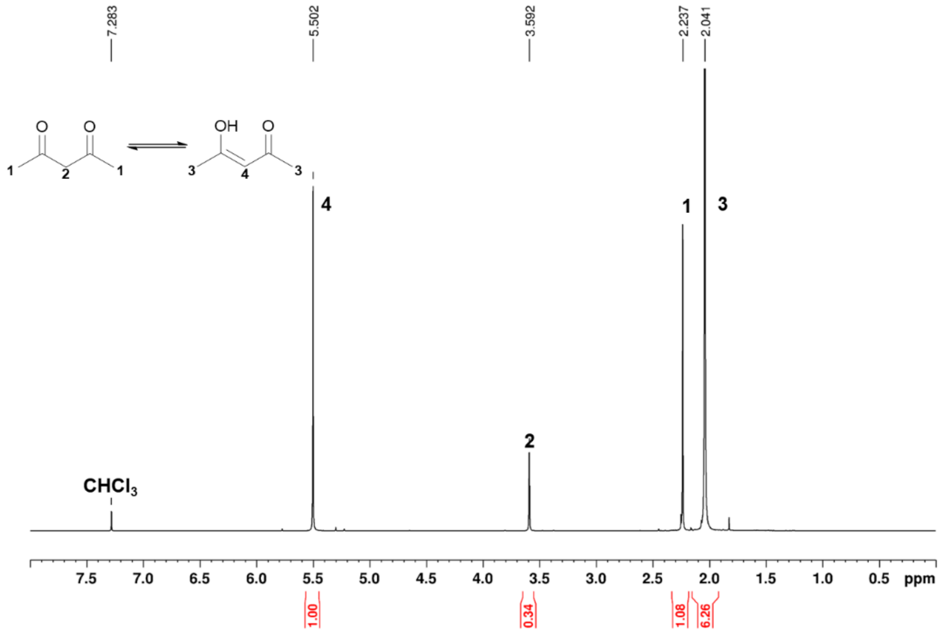

Figure S29: ^{1}H NMR spectrum of acetylacetone with signal assignments (300 MHz, $CDCl_3$). Proton signal assignment based on literature reports.[18]

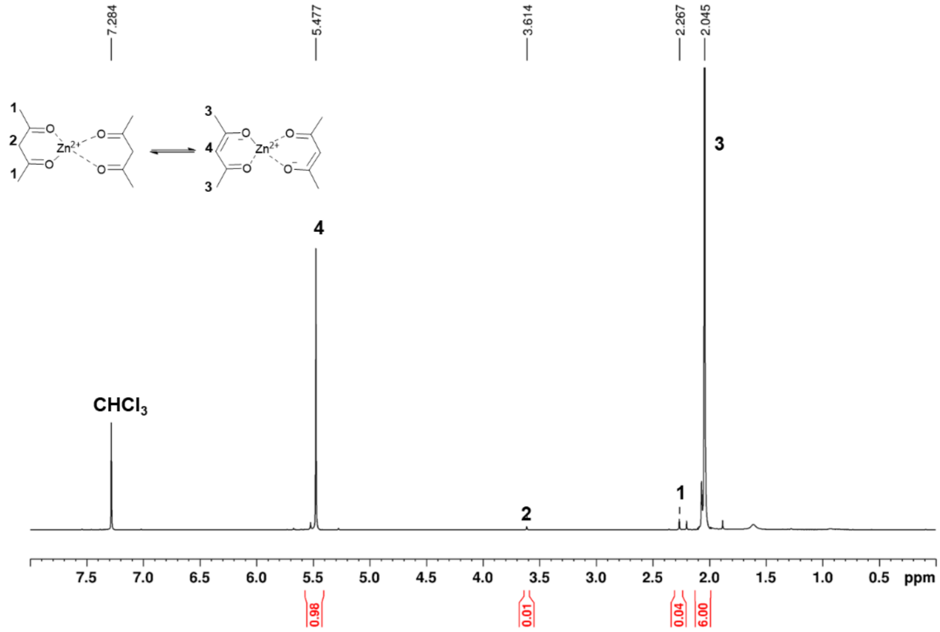

Figure S30: ^{1}H NMR spectrum of *bis*(2,4-pentanedionato) zinc(II) with signal assignments (400 MHz, $CDCl_3$). Proton signal assignment based on literature reports.[18]

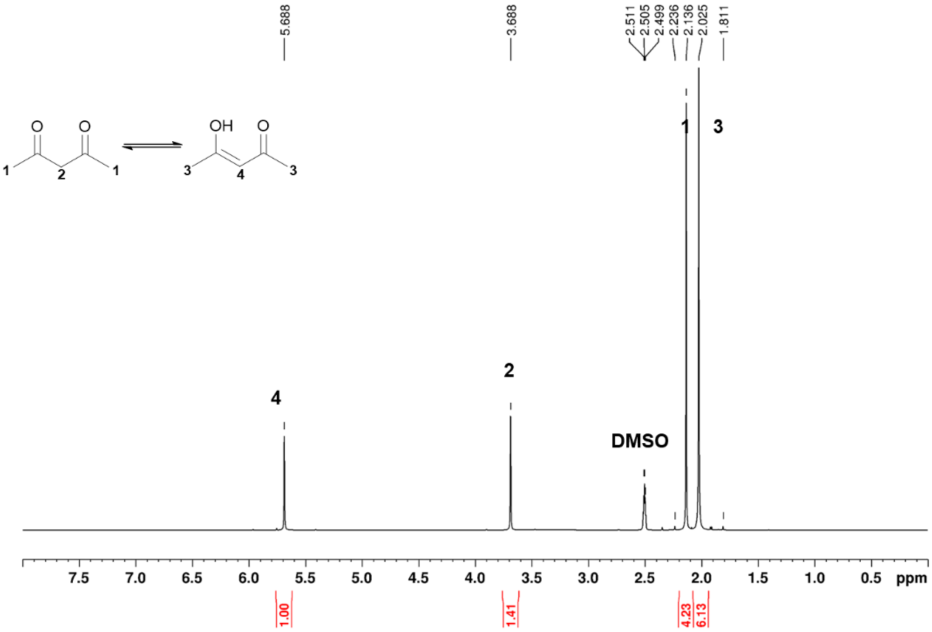

Figure S31: ^{1}H NMR spectrum of acetylacetone with signal assignments (300 MHz, DMSO-*d6*). Proton signal assignment based on literature reports.[18]

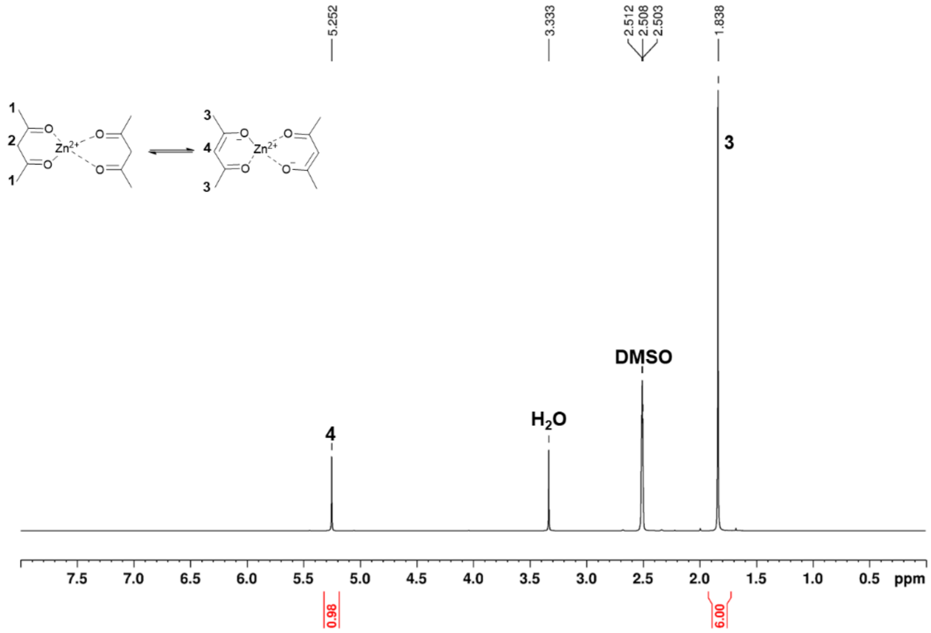

Figure S32: ^{1}H NMR spectrum of *bis*(2,4-pentanedionato) zinc(II) with signal assignments (400 MHz, DMSO-*d6*). Proton signal assignment based on literature reports.[18]

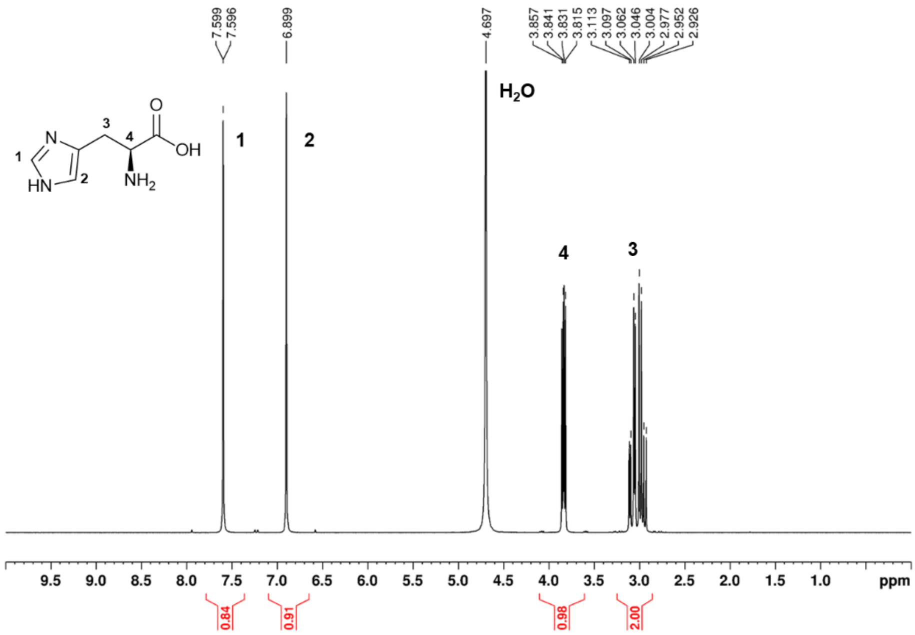

Figure S33: ^{1}H NMR spectrum of *L*-histidine with signal assignments (300 MHz, D_2O). Proton signal assignment based on literature reports.[19]

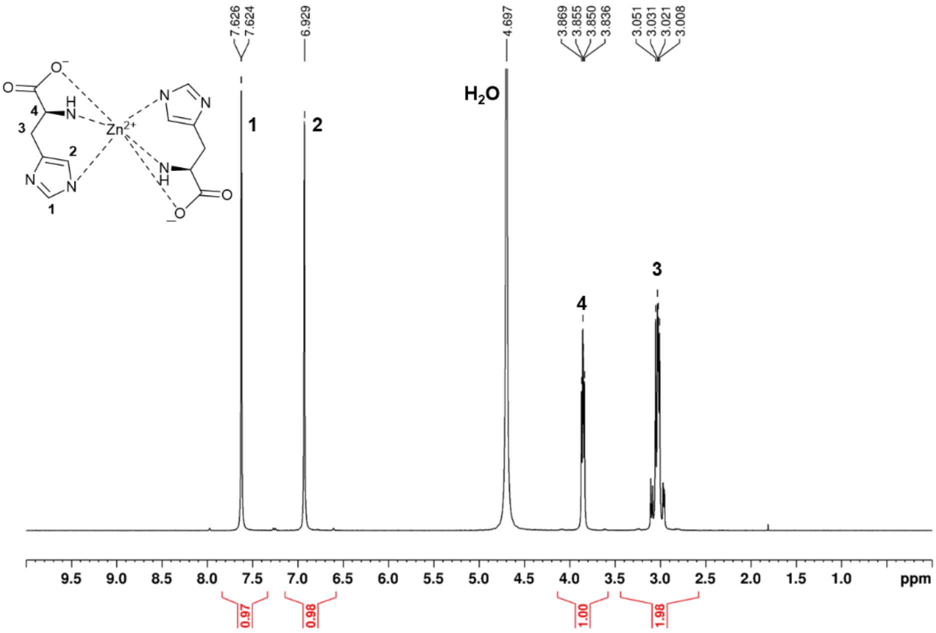

Figure S34: ^{1}H NMR spectrum of a complex of *L*-histidine with zinc(II) carbonate with signal assignments (300 MHz, D_2O). Proton signal assignment based on literature reports.[19]

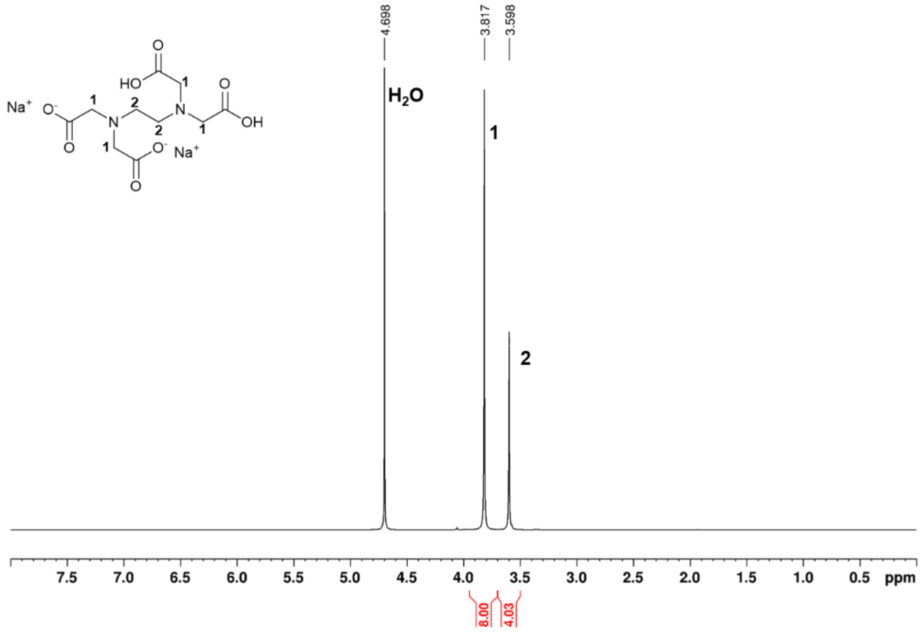

Figure S35: ^{1}H NMR spectrum of ethylenediaminetetraacetic acid disodium salt with signal assignments (300 MHz, D_2O). Proton signal assignment based on literature reports.[20]

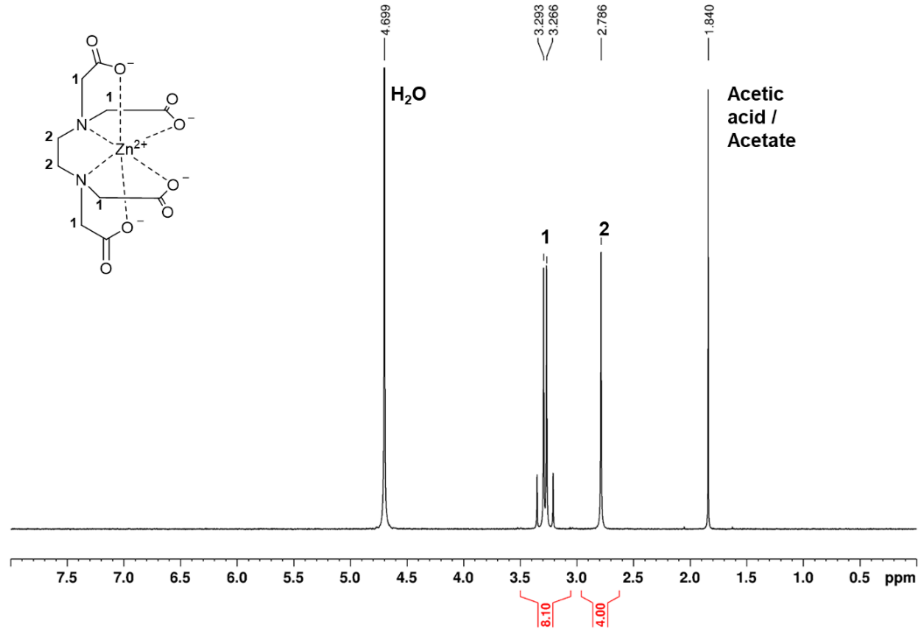

Figure S36: ^{1}H NMR spectrum of a complex of the ethylenediaminetetraacetic acid disodium salt with zinc(II) acetate with signal assignments (300 MHz, D_2O). Proton signal assignment based on literature reports.[20]

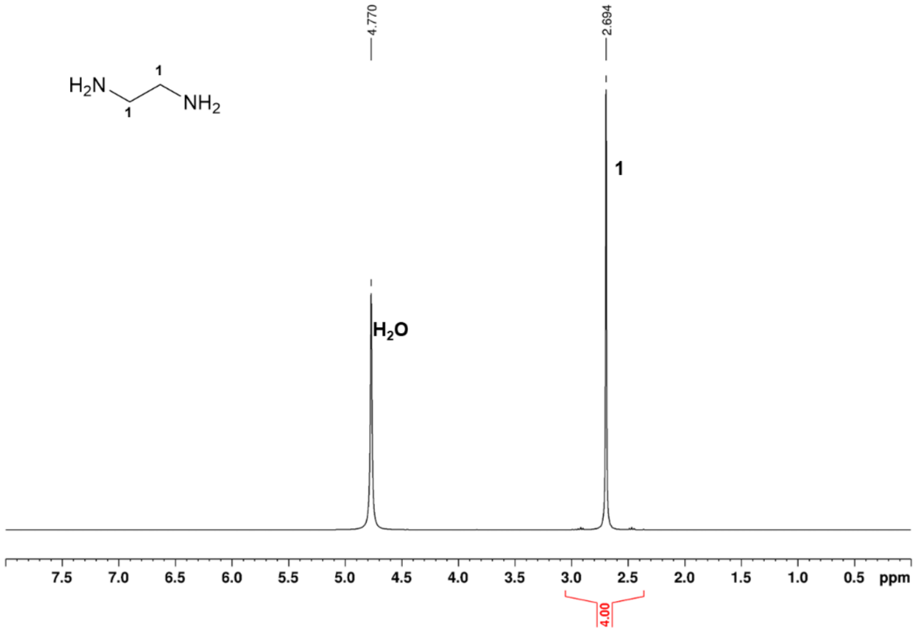

Figure S37: ^{1}H NMR spectrum of ethylenediamine with signal assignments (300 MHz, D_2O).

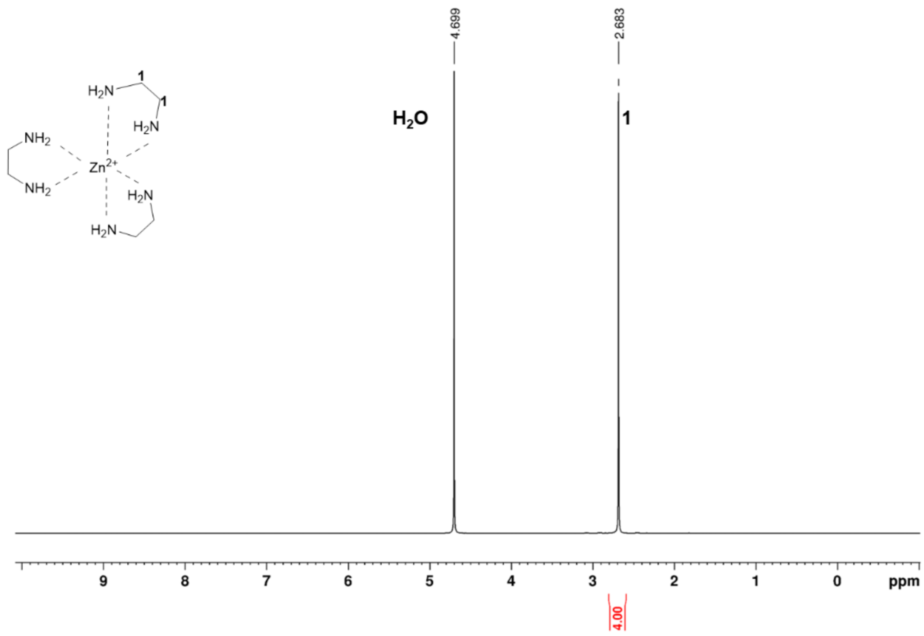

Figure S38: ^{1}H NMR spectrum of a complex of ethylenediamine with zinc(II) sulphate with signal assignments (300 MHz, D_2O).

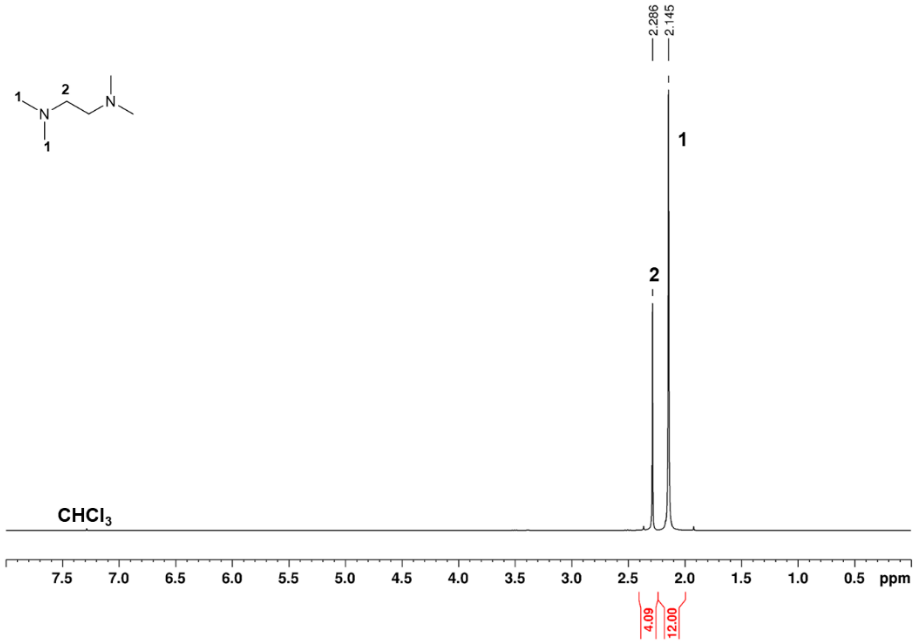

Figure S39: ^{1}H NMR spectrum of *N,N,N',N'*-tetramethyl ethylenediamine with signal assignments (300 MHz, $CDCl_3$).

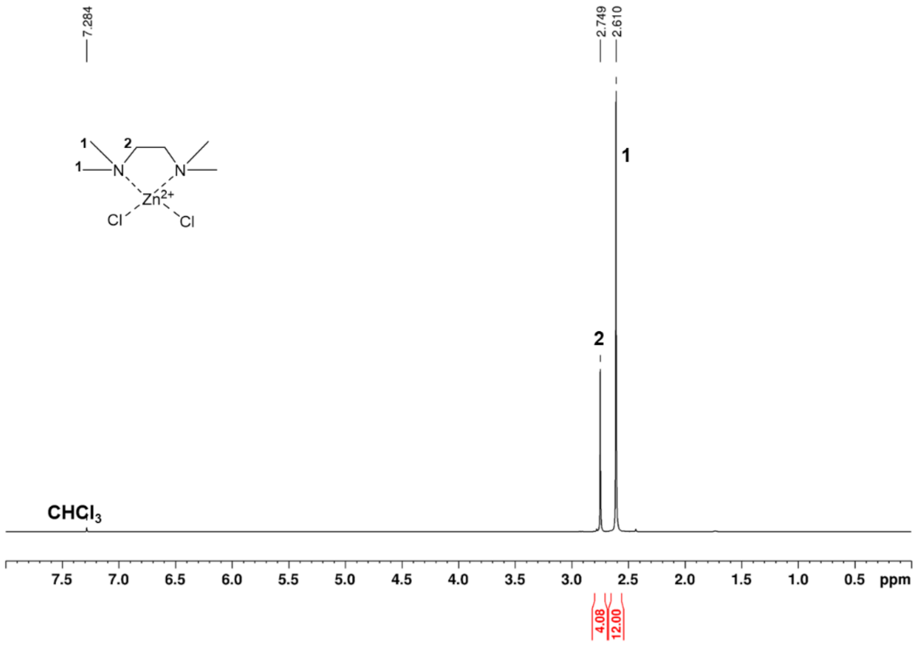

Figure S40: ^{1}H NMR spectrum of dichloro (*N,N,N',N'*-tetramethyl ethylenediamine) zinc(II) with signal assignments (400 MHz, $CDCl_3$).

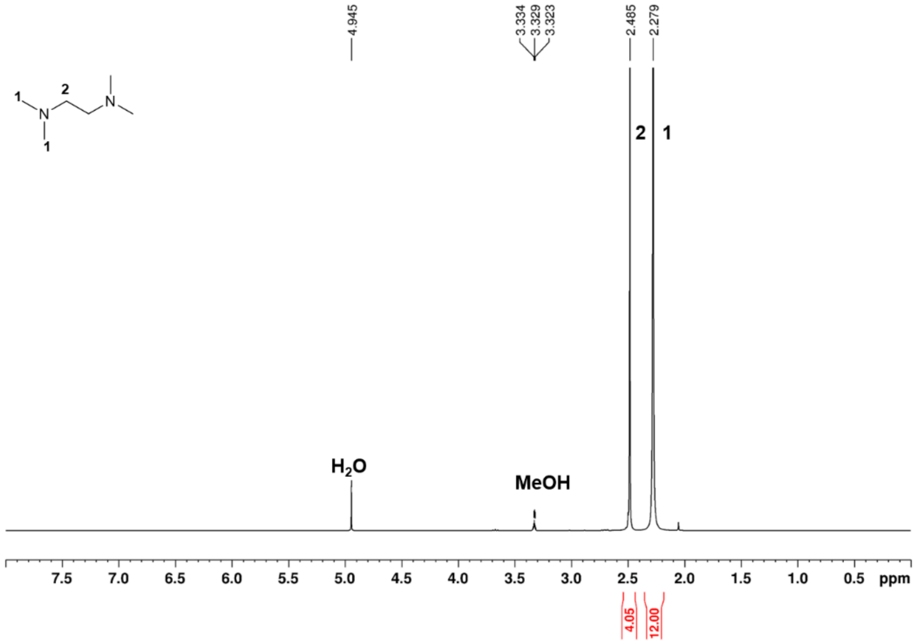

Figure S41: ^{1}H NMR spectrum of *N,N,N′,N′*-tetramethyl ethylenediamine with signal assignments (300 MHz, MeOH-*d4*).

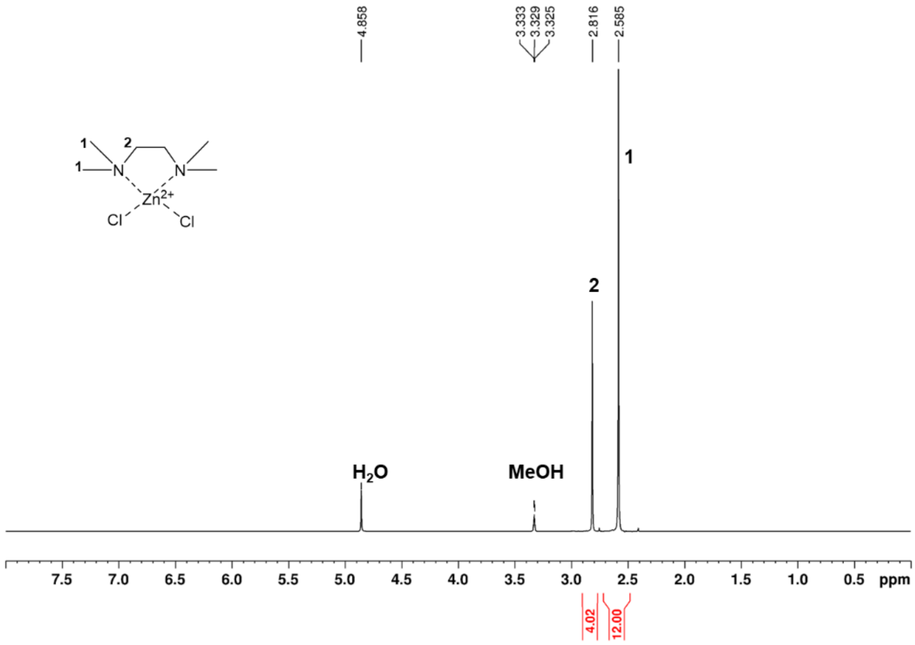

Figure S42: ^{1}H NMR spectrum of dichloro (*N,N,N′,N′*-tetramethyl ethylenediamine) zinc(II) with signal assignments (400 MHz, MeOH-*d4*).

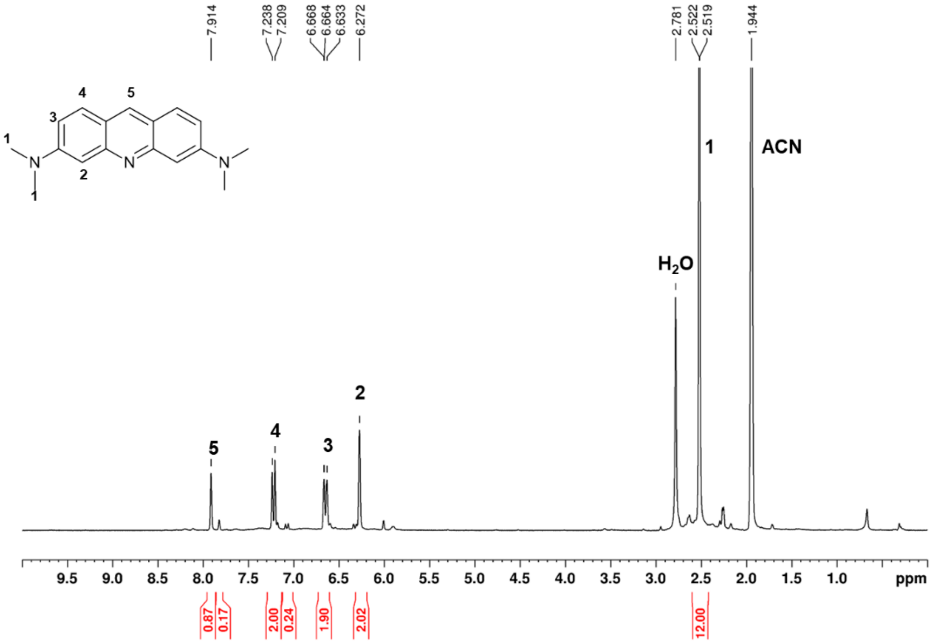

Figure S43: ^{1}H NMR spectrum of *N,N,N′,N′*-tetramethylacridine-3,6-diamine (acridine orange, free base) with signal assignments (300 MHz, ACN-*d3*). Proton signal assignment based on peak integral values and literature reports.[21]

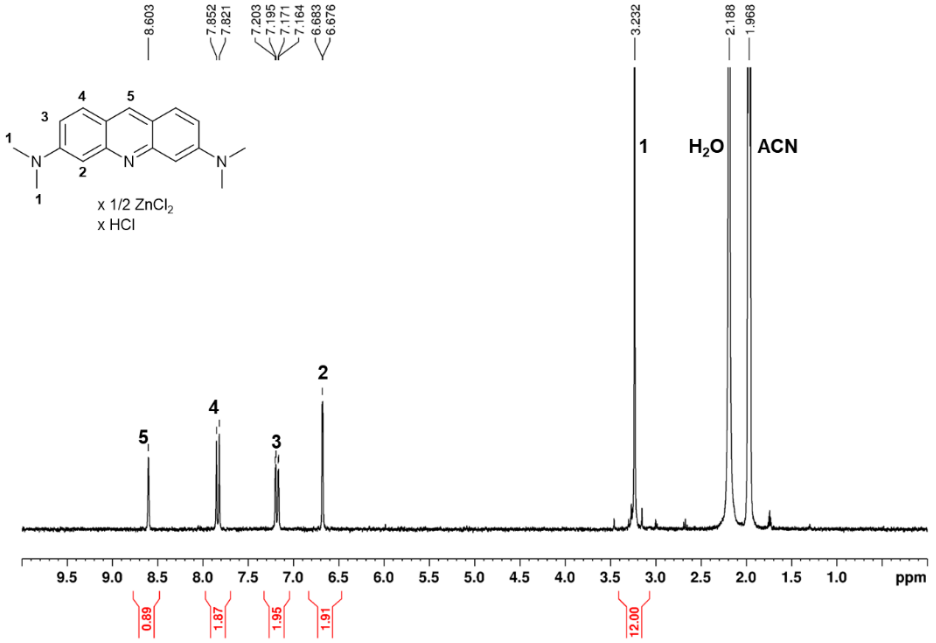

Figure S44: ^{1}H NMR spectrum of *N,N,N′,N′*-tetramethylacridin-3,6-diamine hemizinc salt (acridine orange hemi (zinc(II) chloride) salt) with signal assignments (300 MHz, ACN-*d3*). Proton signal assignment based on peak integral values and literature reports.[21]

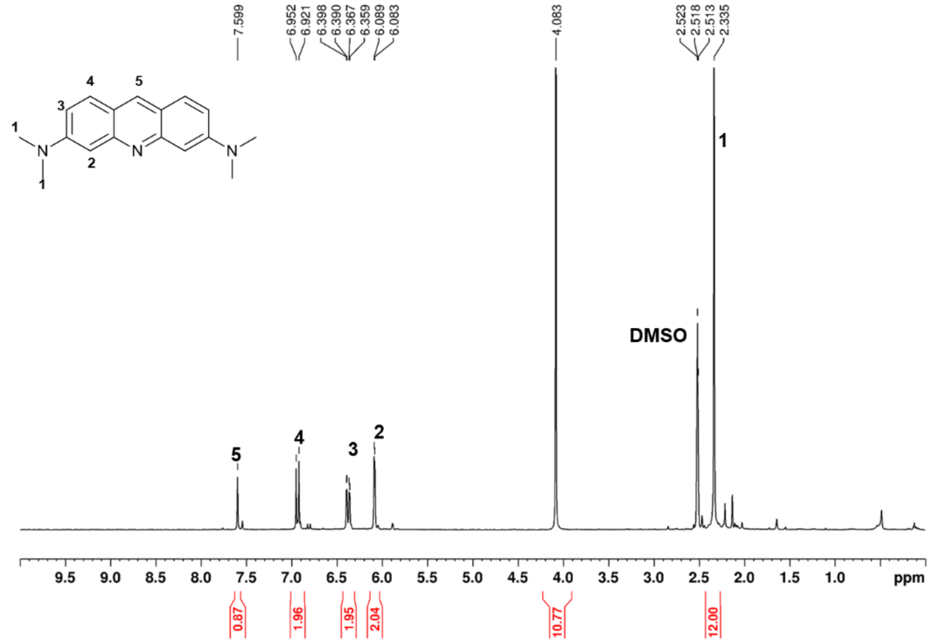

Figure S45: ^{1}H NMR spectrum of *N,N,N',N'*-tetramethylacridine-3,6-diamine (acridine orange, free base) with signal assignments (300 MHz, DMSO-*d6*). Proton signal assignment based on peak integral values and literature reports.[21]

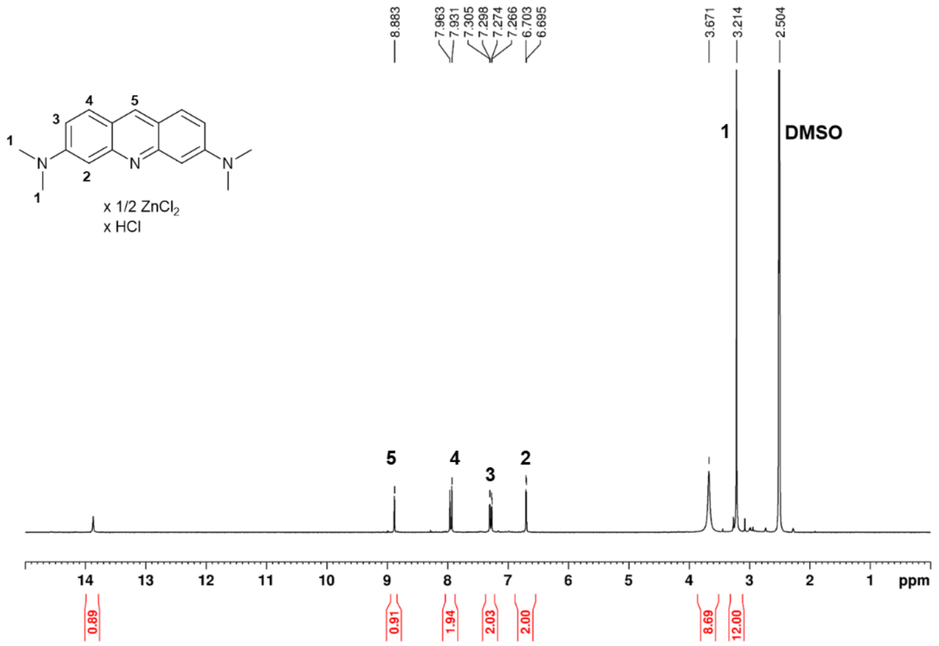

Figure S46: ^{1}H NMR spectrum of *N,N,N',N'*-tetramethylacridine-3,6-diamine hemizinc salt (acridine orange hemi (zinc(II) chloride) salt) with signal assignments (300 MHz, DMSO-*d6*). Proton signal assignment based on peak integral values and literature reports.[21]

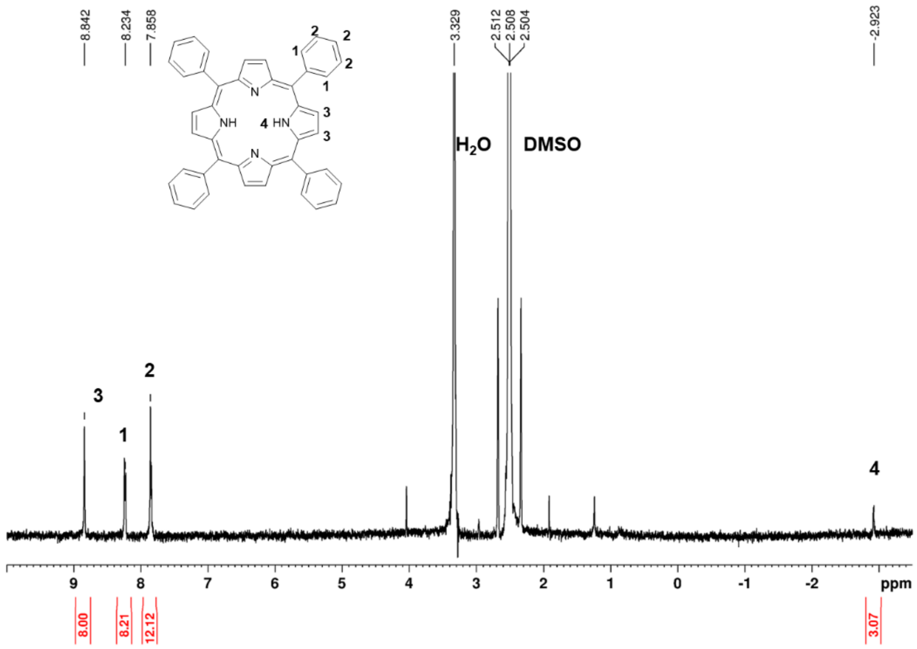

Figure S47: ^{1}H NMR spectrum of tetraphenylporphyrin with signal assignments (400 MHz, DMSO-*d6*). Proton signal assignment based on peak integral values and literature reports.[22]

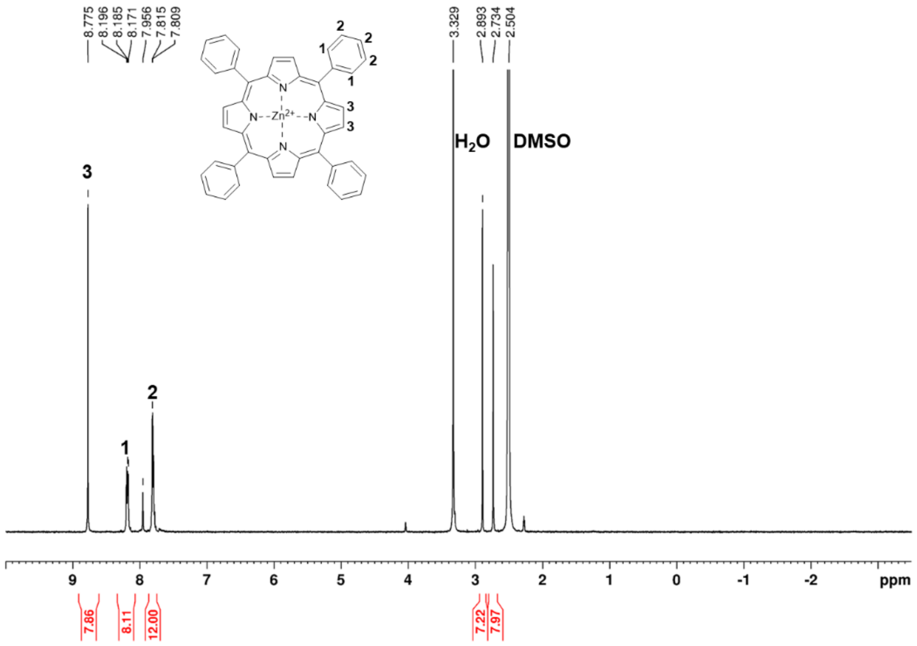

Figure S48: ^{1}H NMR spectrum of 5,10,15,20-tetraphenyl-21H,23H-porphine zinc(II) with signal assignments (300 MHz, DMSO-*d6*). Proton signal assignment based on peak integral values and literature reports.[22]

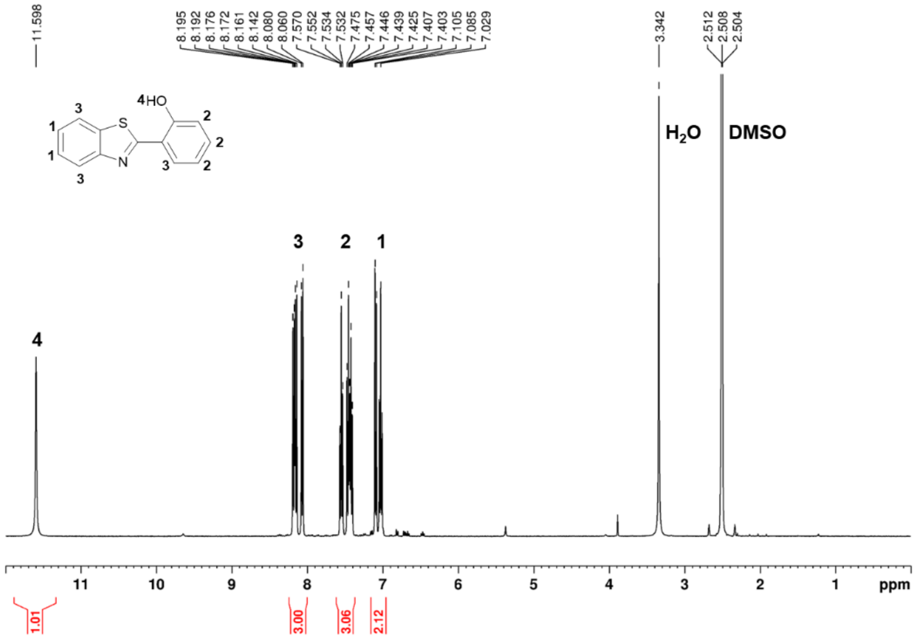

Figure S49: ^{1}H NMR spectrum of 2-(2-hydroxyphenyl) benzothiazole with signal assignments (400 MHz, DMSO-*d6*). Proton signal assignment based on peak integral values and literature reports.[23]

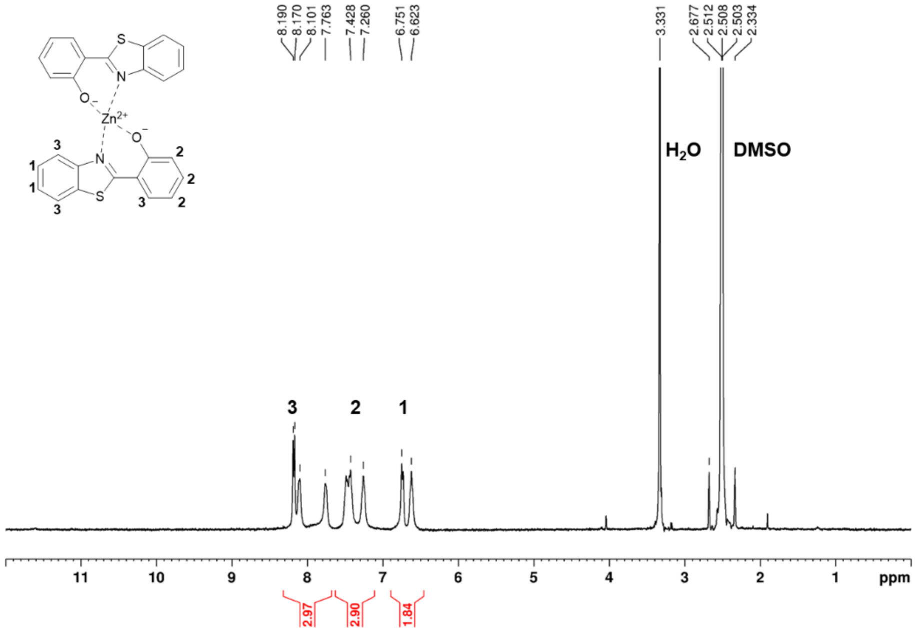

Figure S50: ^{1}H NMR spectrum of bis[2-(2-benzothiazolyl) phenolato] zinc(II) with signal assignments (400 MHz, DMSO-*d6*). Proton signal assignment based on peak integral values and literature reports.[23]

5. References

1. Balcells, D.; Skjelstad, B. B. tmQM dataset—quantum geometries and properties of 86k transition metal complexes. Journal of Chemical Information and Modeling **2020**, 60, 6135–6146.
2. Bartók, A. P.; Kondor, R.; Csányi, G. On representing chemical environments. Physical Review B—Condensed Matter and Materials Physics **2013**, 87, 184115.
3. Ahmed, M.; Seraj, R.; Islam, S. M. S. The k-means algorithm: A comprehensive survey and performance evaluation. Electronics **2020**, 9, 1295.
4. Allen, F. H.; Taylor, R. Research applications of the Cambridge structural database (CSD). Chemical Society Reviews **2004**, 33, 463-475.
5. Himanen, L.; Jäger, M. O.; Morooka, E. V.; Canova, F. F.; Ranawat, Y. S.; Gao, D.; Z., Rinke, Patrick.;Foster, A. S. DScribe: Library of descriptors for machine learning in materials science. Computer Physics Communications **2020**, 247, 106949.
6. Pedregosa, F. et al. Scikit-learn: Machine learning in Python. The Journal of Machine Learning Research **2011**, 12, 2825–2830.
7. Ringleb, M.; Klähn, L.; Zechel, S.; Schubert, U. S. Automated investigation of metal-ligand interactions by a newly established robotic workflow for titrations. ChemPlusChem **2025**, 90, e202400686.
8. Ferrer, P.; Jiménez-Villacorta, F., Rubio-Zuazo, J.; Da Silva, I.; Castro, G. R. Environmental influence on Zn–histidine complexes under no-packing conditions. The Journal of Physical Chemistry B **2014**, 118, 2842-2850.
9. Wei, D.; Zhang, F.; Cai, Z.; Zhai, B.; Wang, X.; Song, Y. Zn-ethylenediaminetetraacetic acid complex derived N-doped porous carbon for high-performance supercapacitor. Journal of Energy Storage **2023**, 60, 106659.
10. Brabha, M. J.; Malbi, M. A. Synthesis, characterization and biological activity of zinc complexes of ethylenediamine and its derivatives. Chemical Physics Impact **2023**, 7, 100248.
11. Ruddlesden, A. J.; Mewis, R. E.; Green, G. G.; Whitwood, A. C.; Duckett, S. B. Catalytic transfer of magnetism using a neutral iridium phenoxide complex. *Organometallics* **2015**, 34, 2997-3006.
12. Shen, W. Z.; Trötscher-Kaus, G.; Lippert, B. ^{1}H NMR spectroscopic identification of binding modes of 2, 2′-bipyridine ligands in complexes of square-planar d 8 metal ions. Dalton Transactions **2009**, 39, 8203-8214.
13. Vitvarová, T.; Zedník, J.; Bláha, M.; Vohlídal, J.; Svoboda, J. Effect of Ethynyl and 2-Thienyl Substituents on the Complexation of 4′-Substituted 2, 2′: 6′, 2 ″-Terpyridines with Zn^{2+} and Fe^{2+} Ions, and the Spectroscopic Properties of the Ligands and Formed Complex Species. European Journal of Inorganic Chemistry **2012***,* 24, 3866-3874.

14. Jeżowska-Trzebiatowska, B.; Kozłowski, H.; Latos-Grażyński, L.; Kowalik, T. Nuclear magnetic resonance of Zn (II) complexes with 2, 2′-bipyridine. Chemical Physics Letters **1975**, 30, 355-357.

15. Pow, R. W.; Xuan, W.; Long, D. L.; Bell, N. L.; Cronin, L. Embedding alkenes within an icosahedral inorganic fullerene {(NH 4) 42 [Mo 132 O 372 (L) 30 (H 2 O) 72]} for trapping volatile organics. Chemical Science **2020**, 11, 2388-2393.

16. Blohm, S.; Heinze, T. Mechanistic considerations of efficient esterification of starch with propionic anhydride/lauric acid in the green solvent imidazole. Macromolecular Chemistry and Physics **2020**, 221, 2000264.

17. Olvera-Ureña, E.; Lopez-Tellez, J.; Vizueto, M. M.; Hidalgo-Ledezma, J. G.; Martinez-Quiroz, B.; Rodriguez, J. A. Lipase-assisted synthesis of alkyl stearates: Optimization by Taguchi design of experiments and application as defoamers. Molecules **2023**, 29, 195.

18. Sandusky, P. O. Expansion of the Classic Acetylacetone Physical Chemistry Laboratory NMR Experiment: Correlation of the Enol–Keto Equilibrium Position with the Solvent Dipole Moment. Journal of Chemical Education **2014**, 91, 739-742.

19. Ajikumar, A.; Premkumar, A. K. N.; Narayanan, S. P. The self-assembly of l-histidine might be the cause of histidinemia. Scientific Reports **2023**, 13, 17461.

20. Hafer, E.; Holzgrabe, U.; Kraus, K.; Adams, K.; Hook, J. M.; Diehl, B. Qualitative and quantitative 1H NMR spectroscopy for determination of divalent metal cation concentration in model salt solutions, food supplements, and pharmaceutical products by using EDTA as chelating agent. Magnetic Resonance in Chemistry **2020**, 58, 653-665.

21. Totland, C.; Thomas, P. J.; Holst, B.; Akhtar, N.; Hovdenes, J.; Skodvin, T. The use of surfactant-filled mesoporous silica as an immobilising medium for a fluorescence lifetime pH indicator, providing long-term calibration stability. RSC advances **2019**, 9, 37241-37244.

22. Saltan, F.; Palamutlu, A.; Akat, H. Copolymerization of 5-[4-(methacryloylamino) phenyl]-l0, 15, 20-triphenylporphyrin with Styrene: Investigation of Spectroscopic, Structural and Thermal Properties. Journal of Polymer Materials **2019**, 36, 133-147

23. da Luz, L. C.; Toledo, V. P.; Rodembusch, F. S.; Scorsin, L. NMR-based structural analysis of ESIPT-reactive 2-(2′-hydroxy-3′-methylphenyl) benzazoles and their formyl derivatives. Journal of Molecular Structure **2025**, 1345, 143103.
