# Supplementary material for: Efficient Machine Learning Prediction of Solvent‐Dependent NMR Chemical Shifts in Zinc Complexes
Source: J Comput Chem. 2026 Apr 12;47(10):e70368. doi: 10.1002/jcc.70368 (PMC13071242; doi:10.1002/jcc.70368)
Supplement: Supplementary file 1 — Data S1: Supporting Information. [file JCC-47-0-s001.zip › 1HNMR_SI_Captions.docx]

*Supporting Information – Table and Figure Captions*

*Efficient machine learning prediction of solvent-dependent ÅH NMR*

*chemical shifts in zinc complexes*

**Figure S1**. t-SNE visualization of 5,331 zinc complexes clustered based on SOAP descriptors. The 45 representative structures selected for the training dataset are highlighted with black markers.

**Table S1.** Variance explained by principal components.

**Table S2.** K-S test results comparing selected 45 complexes to full database.

**Figure S2.** Distribution comparison between the full database and a selected subset for principal components PC1-PC10. Histograms show the distribution of PC scores for the full database (gray) and selected 45 complexes (blue). P-values from K-S tests are shown in each panel. Nine of ten components show no significant difference (p > 0.05), confirming representative sampling.

**Figure S3.** Structural diversity comparison. Distribution of the pairwise SOAP descriptor distances for a random sample from the full database (gray) and the 45 selected complexes (blue). The similar distribution widths confirm that the selected set captures the structural diversity of the full database.

**Table S3.** Performance comparison of splitting strategies for LightGBM model.

**Figure S4.** Schematic representation of the structure of the complexes which were experimentally prepared sorted by their binding motifs.

**Figure S5.** 1H NMR spectrum of pyridine in a mixture of deuterated methanol (10 v%) and HPLC-grade methanol (90 v%) with peak assignments (300 MHz). Proton signal assignment based on peak integral values and literature reports [11].

**Figure S6.** 1H NMR spectrum of a complex of pyridine with zinc(II) acetate in a mixture of deuterated methanol (10 v%) and HPLC-grade methanol (90 v%) with peak assignments (300 MHz). Proton signal assignment based on peak integral values and literature reports [11].

**Figure S7.** 1H NMR spectrum of 2,2’-bipyridine in a mixture of deuterated methanol (10 v%) and HPLC-grade methanol (90 v%) with peak assignments (300 MHz). Proton signal assignment based on literature reports [12].

**Figure S8.** 1H NMR spectrum of 2,2’:6’,2’’-terpyridine in a mixture of deuterated methanol (10 v%) and HPLC-grade methanol (90 v%) with peak assignments (300 MHz). Proton signal assignment based on literature reports [13].

**Figure S9.** 1H NMR spectrum of a complex of 2,2’:6’,2’’-terpyridine with zinc(II) acetate in a mixture of deuterated methanol (10 v%) and HPLC-grade methanol (90 v%) with peak assignments (300 MHz). Proton signal assignment based on literature reports [13].

**Figure S10.** 1H NMR spectrum of pyridine in a mixture of deuterated dimethyl sulfoxide (DMSO) (10 v%) and HPLC-grade DMSO (90 v%) and added TMS with peak assignments (300 MHz). Proton signal assignment based on peak integral values and literature reports [11].

**Figure S11.** 1H NMR spectrum of a complex of pyridine with zinc(II) acetate in a mixture of deuterated DMSO (10 v%) and HPLC-grade DMSO (90 v%) and added TMS with peak assignments (300 MHz). Proton signal assignment based on peak integral values and literature reports [11].

**Figure S12.** 1H NMR spectrum of 2,2’-bipyridine in a mixture of deuterated DMSO (10 v%) and HPLC-grade DMSO (90 v%) and added TMS with peaks assignment (300 MHz). Proton signal assignment based on literature reports [12].

**Figure S13.** 1H NMR spectrum of a complex of 2,2’-bipyridine with zinc(II) acetate in a mixture of deuterated DMSO (10 v%) and HPLC-grade DMSO (90 v%) and added TMS with peak assignments (300 MHz). Proton signal assignment based on literature reports [14].

**Figure S14.** 1H NMR spectrum of 2,2’:6’,2’’-terpyridine in a mixture of deuterated DMSO (10 v%) and HPLC-grade DMSO (90 v%) and added TMS with peak assignments (300 MHz). Proton signal assignment based on literature reports [12].

**Figure S15.** 1H NMR spectrum of acetic acid with signal assignments (300 MHz, D2O).

**Figure S16.** 1H NMR spectrum of zinc(II) acetate dihydrate with signal assignments (300 MHz, D2O).

**Figure S17.** 1H NMR spectrum of acetic acid with signal assignments (300 MHz, MeOH-d4).

**Figure S18.** 1H NMR spectrum of zinc(II) acetate dihydrate with signal assignments (300 MHz, MeOH-d4).

**Figure S19.** 1H NMR spectrum of methacrylic acid with signal assignments (300 MHz, CDCl3). Proton signal assignment based on literature reports [15].

**Figure S20.** 1H NMR spectrum of a zinc(II) methacrylate with signal assignments (300 MHz, CDCl3). Proton signal assignment based on literature reports [15].

**Figure S21.** 1H NMR spectrum of methacrylic acid with signal assignments (300 MHz, DMSO-d6). Proton signal assignment based on literature reports [15].

**Figure S22.** 1H NMR spectrum of zinc(II) methacrylate with signal assignments (300 MHz, DMSO-d6). Proton signal assignment based on literature reports [15].

**Figure S23.** 1H NMR spectrum of methacrylic acid with signal assignments (300 MHz, MeOH-d4). Proton signal assignment based on literature reports [15].

**Figure S24.** 1H NMR spectrum of a zinc(II) methacrylate with signal assignments (300 MHz, MeOH-d4). Proton signal assignment based on literature reports [15].

**Figure S25.** 1H NMR spectrum of dodecanoic acid with signal assignments (300 MHz, MeOH-d4). Proton signal assignment based on literature reports [16].

**Figure S26.** 1H NMR spectrum of zinc(II) dodecanoate with signal assignments (400 MHz, MeOH-d4). Proton signal assignment based on literature reports [16].

**Figure S27.** 1H NMR spectrum of stearic acid with signal assignments (300 MHz, CDCl3). Proton signal assignment based on literature reports [17].

**Figure S28.** 1H NMR spectrum of zinc(II) stearate with signal assignments (300 MHz, CDCl3). Proton signal assignment based on literature reports [17].

**Figure S29.** 1H NMR spectrum of acetylacetone with signal assignments (300 MHz, CDCl3). Proton signal assignment based on literature reports [18].

**Figure S30.** 1H NMR spectrum of bis(2,4-pentanedionato) zinc(II) with signal assignments (400 MHz, CDCl3). Proton signal assignment based on literature reports [18].

**Figure S31.** 1H NMR spectrum of acetylacetone with signal assignments (300 MHz, DMSO-d6). Proton signal assignment based on literature reports [18].

**Figure S32.** 1H NMR spectrum of bis(2,4-pentanedionato) zinc(II) with signal assignments (400 MHz, DMSO-d6). Proton signal assignment based on literature reports [18].

**Figure S33.** 1H NMR spectrum of L-histidine with signal assignments (300 MHz, D2O). Proton signal assignment based on literature reports [19].

**Figure S34.** 1H NMR spectrum of a complex of L-histidine with zinc(II) carbonate with signal assignments (300 MHz, D2O). Proton signal assignment based on literature reports [19].

**Figure S35.** 1H NMR spectrum of ethylenediaminetetraacetic acid disodium salt with signal assignments (300 MHz, D2O). Proton signal assignment based on literature reports [20].

**Figure S36.** 1H NMR spectrum of a complex of the ethylenediaminetetraacetic acid disodium salt with zinc(II) acetate with signal assignments (300 MHz, D2O). Proton signal assignment based on literature reports [20].

**Figure S37:** 1H NMR spectrum of ethylenediamine with signal assignments (300 MHz, D2O).

**Figure S38:** 1H NMR spectrum of a complex of ethylenediamine with zinc(II) sulphate with signal assignments (300 MHz, D2O).

**Figure S39.** 1H NMR spectrum of N,N,N',N'-tetramethyl ethylenediamine with signal assignments (300 MHz, CDCl3).

**Figure S40.** 1H NMR spectrum of dichloro (N,N,N′,N′-tetramethyl ethylenediamine) zinc(II) with signal assignments (400 MHz, CDCl3).

**Figure S41.** 1H NMR spectrum of N,N,N',N'-tetramethyl ethylenediamine with signal assignments (300 MHz, MeOH-d4).

**Figure S42.** 1H NMR spectrum of dichloro (N,N,N′,N′-tetramethyl ethylenediamine) zinc(II) with signal assignments (400 MHz, MeOH-d4).

**Figure S43.** 1H NMR spectrum of N,N,N′,N′-tetramethylacridine-3,6-diamine (acridine orange, free base) with signal assignments (300 MHz, ACN-d3). Proton signal assignment based on peak integral values and literature reports [21].

**Figure S44.** 1H NMR spectrum of N,N,N′,N′-tetramethylacridin-3,6-diamine hemizinc salt (acridine orange hemi (zinc(II) chloride) salt) with signal assignments (300 MHz, ACN-d3). Proton signal assignment based on peak integral values and literature reports [21].

**Figure S45.** 1H NMR spectrum of N,N,N′,N′-tetramethylacridine-3,6-diamine (acridine orange, free base) with signal assignments (300 MHz, DMSO-d6). Proton signal assignment based on peak integral values and literature reports [21].

**Figure S46.** 1H NMR spectrum of N,N,N′,N′-tetramethylacridine-3,6-diamine hemizinc salt (acridine orange hemi (zinc(II) chloride) salt) with signal assignments (300 MHz, DMSO-d6). Proton signal assignment based on peak integral values and literature reports [21].

**Figure S47.** 1H NMR spectrum of tetraphenylporphyrin with signal assignments (400 MHz, DMSO-d6). Proton signal assignment based on peak integral values and literature reports [22].

**Figure S48.** 1H NMR spectrum of 5,10,15,20-tetraphenyl-21H,23H-porphine zinc(II) with signal assignments (300 MHz, DMSO-d6). Proton signal assignment based on peak integral values and literature reports [22].

**Figure S49.** 1H NMR spectrum of 2-(2-hydroxyphenyl) benzothiazole with signal assignments (400 MHz, DMSO-d6). Proton signal assignment based on peak integral values and literature reports [23].

**Figure S50.** 1H NMR spectrum of bis[2-(2-benzothiazolyl) phenolato] zinc(II) with signal assignments (400 MHz, DMSO-d6). Proton signal assignment based on peak integral values and literature reports [23].

References

1. Balcells, D.; Skjelstad, B. B. tmQM dataset—quantum geometries and properties

of 86k transition metal complexes. Journal of Chemical Information and Modeling

2020, 60, 6135–6146.

2. Bartók, A. P.; Kondor, R.; Csányi, G. On representing chemical environments.

Physical Review B—Condensed Matter and Materials Physics 2013, 87, 184115.

3. Ahmed, M.; Seraj, R.; Islam, S. M. S. The k-means algorithm: A comprehensive

survey and performance evaluation. Electronics 2020, 9, 1295.

4. Allen, F. H.; Taylor, R. Research applications of the Cambridge structural database

(CSD). Chemical Society Reviews 2004, 33, 463-475.

5. Himanen, L.; Jäger, M. O.; Morooka, E. V.; Canova, F. F.; Ranawat, Y. S.; Gao, D.;

Z., Rinke, Patrick.;Foster, A. S. DScribe: Library of descriptors for machine learning

in materials science. Computer Physics Communications 2020, 247, 106949.

6. Pedregosa, F. et al. Scikit-learn: Machine learning in Python. The Journal of

Machine Learning Research 2011, 12, 2825–2830.

7. Ringleb, M.; Klähn, L.; Zechel, S.; Schubert, U. S. Automated investigation of metalligand

interactions by a newly established robotic workflow for titrations.

ChemPlusChem 2025, 90, e202400686.

8. Ferrer, P.; Jiménez-Villacorta, F., Rubio-Zuazo, J.; Da Silva, I.; Castro, G. R.

Environmental influence on Zn–histidine complexes under no-packing conditions.

The Journal of Physical Chemistry B 2014, 118, 2842-2850.

9. Wei, D.; Zhang, F.; Cai, Z.; Zhai, B.; Wang, X.; Song, Y. Znethylenediaminetetraacetic

acid complex derived N-doped porous carbon for highperformance

supercapacitor. Journal of Energy Storage 2023, 60, 106659.

10. Brabha, M. J.; Malbi, M. A. Synthesis, characterization and biological activity of

zinc complexes of ethylenediamine and its derivatives. Chemical Physics Impact

2023, 7, 100248.

11. Ruddlesden, A. J.; Mewis, R. E.; Green, G. G.; Whitwood, A. C.; Duckett, S. B.

Catalytic transfer of magnetism using a neutral iridium phenoxide

complex. Organometallics 2015, 34, 2997-3006.

12. Shen, W. Z.; Trötscher-Kaus, G.; Lippert, B. 1H NMR spectroscopic identification of

binding modes of 2, 2′-bipyridine ligands in complexes of square-planar d 8 metal

ions. Dalton Transactions 2009, 39, 8203-8214.

13. Vitvarová, T.; Zedník, J.; Bláha, M.; Vohlídal, J.; Svoboda, J. Effect of Ethynyl and

2-Thienyl Substituents on the Complexation of 4′-Substituted 2, 2′: 6′, 2 ″-

Terpyridines with Zn2+ and Fe2+ Ions, and the Spectroscopic Properties of the

Ligands and Formed Complex Species. European Journal of Inorganic

Chemistry 2012, 24, 3866-3874.

38

14. Jeżowska-Trzebiatowska, B.; Kozłowski, H.; Latos-Grażyński, L.; Kowalik, T.

Nuclear magnetic resonance of Zn (II) complexes with 2, 2′-bipyridine. Chemical

Physics Letters 1975, 30, 355-357.

15. Pow, R. W.; Xuan, W.; Long, D. L.; Bell, N. L.; Cronin, L. Embedding alkenes within

an icosahedral inorganic fullerene {(NH 4) 42 [Mo 132 O 372 (L) 30 (H 2 O) 72]} for

trapping volatile organics. Chemical Science 2020, 11, 2388-2393.

16. Blohm, S.; Heinze, T. Mechanistic considerations of efficient esterification of starch

with propionic anhydride/lauric acid in the green solvent imidazole. Macromolecular

Chemistry and Physics 2020, 221, 2000264.

17. Olvera-Ureña, E.; Lopez-Tellez, J.; Vizueto, M. M.; Hidalgo-Ledezma, J. G.;

Martinez-Quiroz, B.; Rodriguez, J. A. Lipase-assisted synthesis of alkyl stearates:

Optimization by Taguchi design of experiments and application as

defoamers. Molecules 2023, 29, 195.

18. Sandusky, P. O. Expansion of the Classic Acetylacetone Physical Chemistry

Laboratory NMR Experiment: Correlation of the Enol–Keto Equilibrium Position

with the Solvent Dipole Moment. Journal of Chemical Education 2014, 91, 739-742.

19. Ajikumar, A.; Premkumar, A. K. N.; Narayanan, S. P. The self-assembly of l-histidine

might be the cause of histidinemia. Scientific Reports 2023, 13, 17461.

20. Hafer, E.; Holzgrabe, U.; Kraus, K.; Adams, K.; Hook, J. M.; Diehl, B. Qualitative

and quantitative 1H NMR spectroscopy for determination of divalent metal cation

concentration in model salt solutions, food supplements, and pharmaceutical

products by using EDTA as chelating agent. Magnetic Resonance in Chemistry

2020, 58, 653-665.

21. Totland, C.; Thomas, P. J.; Holst, B.; Akhtar, N.; Hovdenes, J.; Skodvin, T. The use

of surfactant-filled mesoporous silica as an immobilising medium for a fluorescence

lifetime pH indicator, providing long-term calibration stability. RSC advances 2019,

9, 37241-37244.

22. Saltan, F.; Palamutlu, A.; Akat, H. Copolymerization of 5-[4-(methacryloylamino)

phenyl]-l0, 15, 20-triphenylporphyrin with Styrene: Investigation of Spectroscopic,

Structural and Thermal Properties. Journal of Polymer Materials 2019, 36, 133-147

23. da Luz, L. C.; Toledo, V. P.; Rodembusch, F. S.; Scorsin, L. NMR-based structural

analysis of ESIPT-reactive 2-(2′-hydroxy-3′-methylphenyl) benzazoles and their

formyl derivatives. Journal of Molecular Structure 2025, 1345, 143103.
